# Inhibition of AhR improves cortical bone and skeletal muscle function via preservation of neuromuscular junctions

**DOI:** 10.1172/jci.insight.192047

**Published:** 2025-07-15

**Authors:** Kanglun Yu, Sagar Vyavahare, Dima Alhamad, Husam Bensreti, Ling Ruan, Anik Tuladhar, Caihong Dai, Joseph C. Shaver, Alok Tripathi, Kehong Ding, Rafal Pacholczyk, Marion A. Cooley, Roger Zhong, Maribeth H. Johnson, Jie Chen, Wendy B. Bollag, Carlos M. Isales, William D. Hill, Mark W. Hamrick, Sadanand Fulzele, Meghan E. McGee-Lawrence

**Affiliations:** 1 Department of Cellular Biology and Anatomy, Medical College of Georgia,; 2 Department of Medicine, Medical College of Georgia,; 3 Department of Neuroscience & Regenerative Medicine, Medical College of Georgia,; 4 Georgia Cancer Center,; 5 Department of Oral Biology & Diagnostic Sciences, Dental College of Georgia,; 6 Department Biostatistics, Data Science & Epidemiology, School of Public Health, and; 7 Department of Physiology, Medical College of Georgia, Augusta University, Augusta, Georgia, USA.; 8 Charlie Norwood VA Medical Center, Augusta, Georgia, USA.; 9 Department of Pathology, Medical University of South Carolina, Charleston, South Carolina, USA.

**Keywords:** Aging, Bone biology, Osteoclast/osteoblast biology, Osteoporosis, Skeletal muscle

## Abstract

The aryl hydrocarbon receptor (AhR) is proposed to mediate the frailty-promoting effects of the tryptophan metabolite kynurenine, which increases with age in mice and humans. The goal of the current study was to test whether administration of pharmacological AhR inhibitors, BAY2416964 and CH-223191, could abrogate musculoskeletal decline in aging mice. Female C57BL/6 mice (18 months old) were treated with vehicle (VEH) or 30 mg/kg BAY2416964 (BAY) via daily oral gavage 5 days/week for 8 weeks. A second AhR antagonist, CH-223191, was administered to 16-month-old male and female C57BL/6 mice via intraperitoneal injections (3.3 mg/kg) 3 days/week for 12 weeks. While grip strength declined over time in VEH-treated mice, BAY preserved grip strength in part by improving integrity of neuromuscular junctions (NMJs), an effect replicated during in vitro studies with siRNA against AhR. Cortical bone mass was also greater in BAY- than VEH-treated mice. Similarly, CH-223191 treatment improved cortical bone and showed beneficial effects in skeletal muscle, including reducing oxidative stress as compared with VEH-treated animals. Transcriptomic and proteomic data from BAY-treated mice supported a positive impact of BAY on molecular targets that affect NMJ function. Taken together, these data support AhR as a therapeutic target for improving musculoskeletal health during aging.

## Abstract 2

Drugs that inhibit the aryl hydrocarbon receptor (AhR) improved cortical bone mass and skeletal muscle function in aged mice

## Graphical abstract

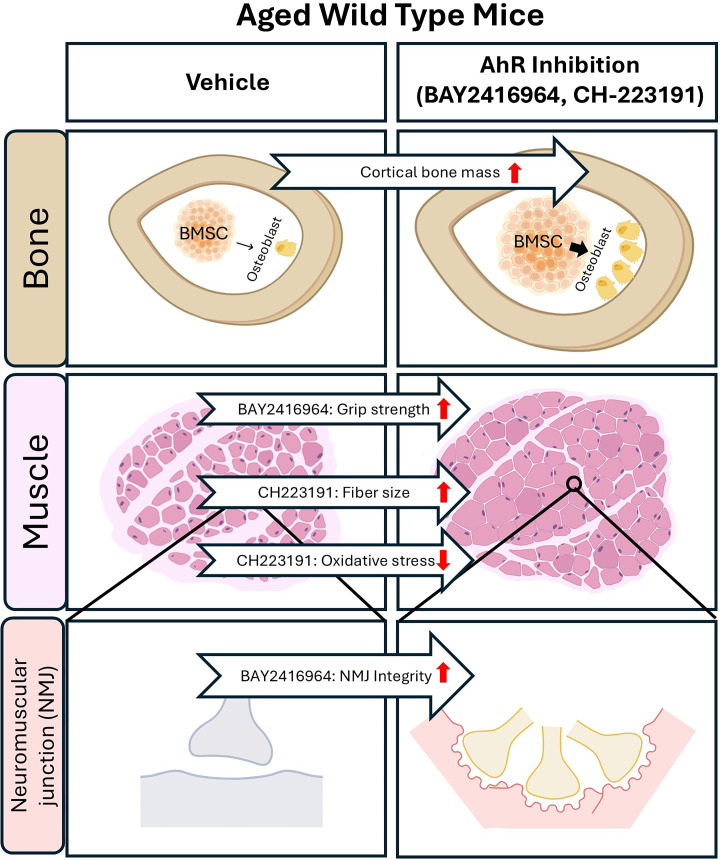

## Introduction

The aryl hydrocarbon receptor (AhR) is a nuclear hormone receptor proposed to mediate effects of the tryptophan metabolite kynurenine and other ligands (1–4). We and others have shown that kynurenine increases with age in mice and humans (5–7), and importantly is associated with musculoskeletal frailty in humans (8–10). Beyond correlative studies, kynurenine has been identified as a likely causative factor for musculoskeletal decline, as several independent studies have demonstrated that administration of exogenous kynurenine promoted phenotypic changes, including muscle atrophy, bone marrow stromal cell (BMSC) senescence, osteoblast and osteoclast dysfunction, and bone loss that together mimic a musculoskeletal aging phenotype (2, 6, 11–15). This raises the possibility that therapeutically targeting the AhR could be beneficial for musculoskeletal health.

While whole-body and tissue-targeted genetic models are an excellent method to mechanistically define the role of AhR in signaling pathways (16–18), pharmacological inhibitors are important from a translational perspective. Several AhR inhibitors are in various stages of clinical development (3). The classical AhR antagonist 2-methyl-2*H*-pyrazole-3-carboxylic acid (CH-223191) was developed in 2006 and has been utilized extensively as an AhR-targeting agent in the years since (3, 19, 20). An AhR inhibitor called BAY2416964 (BAY), an orally active antagonist of AhR, has been more recently developed as an anticancer agent (3, 21). Recent results from clinical trials (ClinicalTrials.gov NCT04069026) suggest effective targeting of AhR by BAY in vivo as well as encouraging preliminary evidence of antitumor activity (22), and a very recent report suggested that BAY efficiently inhibits the AhR in mesenchymal lineage cells (23). The goal of the current study was to test whether pharmacological antagonism of AhR could abrogate musculoskeletal decline in aging mice.

## Results

### BAY inhibited activation of AhR.

**Figure 1:**
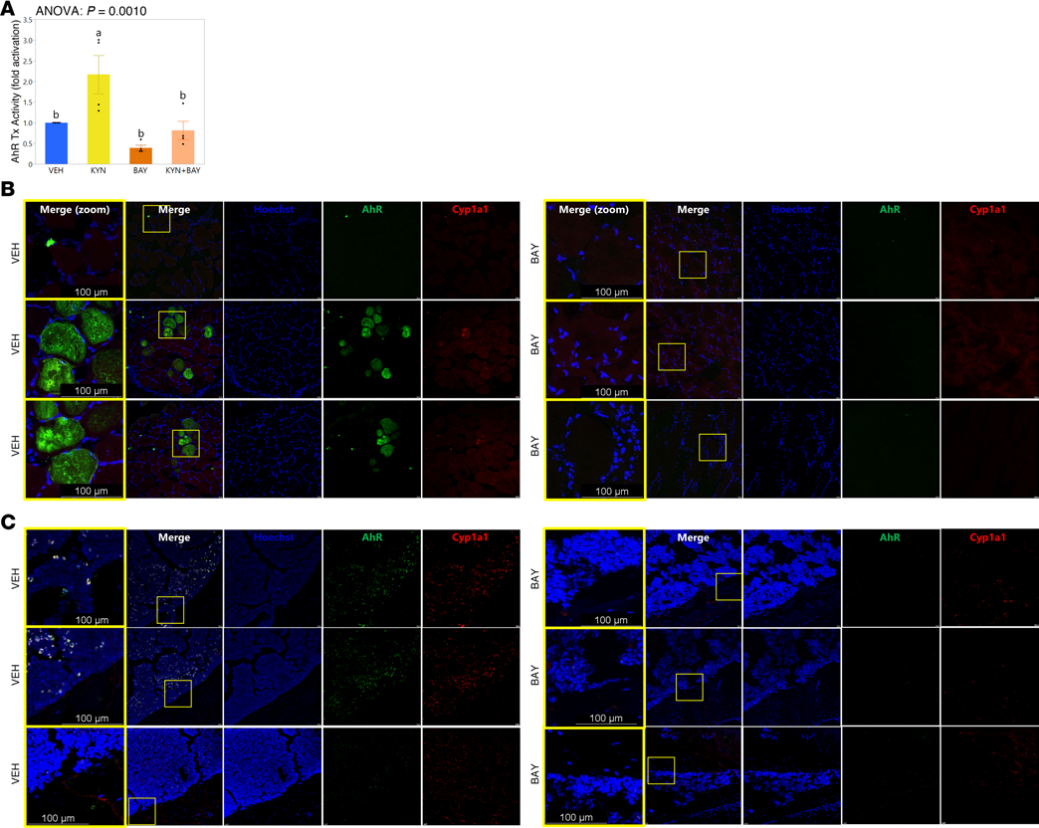
BAY2416964 is an effective AhR antagonist in musculoskeletal cells and tissues. (**A**) AhR transcriptional activity, as shown by a luciferase-based reporter, is activated by kynurenine (KYN; 10 μM) in mesenchymal-lineage ST2 cells, but this impact is blunted by cotreatment with BAY2416964 (BAY); the BAY used in these studies was from the same batch of drug used later for in vivo studies. Group means ± SEM are shown; 1-way ANOVA with Fisher’s LSD post hoc analysis *P* values are shown above the graph, and groups with different superscript letters are significantly (*P* < 0.05) different from one another as shown by post hoc testing (**B** and **C**) RNAscope staining of AhR and Cyp1a1 mRNA expression show effective suppression of these genes in (**B**) TA and (**C**) tibias of BAY- as compared with VEH-treated mice. A magnified image, as demarcated in the yellow box in the columns of images labeled “Merge,” is provided in the columns of images labeled “Merge (zoom)” with scale bars indicative of image size.

Prior to beginning in vivo studies, we tested the efficacy of BAY as an AhR antagonist. We previously showed that kynurenine activates AhR transcriptional activity in mesenchymal lineage ST2 cells as shown by activation of a luciferase reporter featuring xenobiotic response element (XRE) binding sites for AhR and upregulation of AhR target genes, including Cyp1a1 (15). In the current study, induction of AhR transcriptional activity by 10 μM kynurenine in ST2 cells was effectively inhibited by cotreatment with BAY (Figure 1A). Moreover, we conducted RNAscope analyses of skeletal muscle and bone in the aged female BAY- and vehicle-treated (VEH-treated) mice (described below) to investigate expression of AhR and its target gene Cyp1a1, demonstrating that expression of these genes was suppressed in BAY-treated as compared with VEH-treated mice (Figure 1, B and C). BAY therefore appears to effectively inhibit AhR in musculoskeletal tissues.

### BAY treatment did not affect body mass or whole body composition but promoted modest decreases in skeletal muscle mass for fast-twitch muscles.

In preliminary studies (Supplemental Methods; supplemental material available online with this article; https://doi.org/10.1172/jci.insight.192047DS1), we delivered BAY or VEH to young (16-week-old) male and female C57BL/6 mice for 4 weeks (Supplemental Figure 1). While both female and male BAY-treated mice showed increased muscle endurance, as measured by hang time, compared with VEH-treated mice (Supplemental Figure 1, A–C), grip strength was only improved in BAY-treated females (Supplemental Figure 1, D and E). In addition, expression of the AhR target gene Cyp1a1 was significantly higher in the tibialis anterior (TA) from aged (22-month-old) as compared with young (4-month-old) untreated mice (Supplemental Methods), suggesting elevated AhR signaling in muscle with age. These findings suggested that effects of AhR inhibition may be more pronounced in an aged model. We therefore pursued further studies in aged mice focusing on female animals. When we delivered BAY via intraperitoneal injection in preliminary studies, we noted varying degrees of precipitation of the drug in the peritoneal cavity during necropsy (Supplemental Figure 1G; note yellow arrows). We therefore altered the route of delivery to oral gavage for the remaining studies to address solubility concerns.

In the aged mouse studies, neither body mass nor body composition (%fat, %lean) were different between groups at baseline, prior to the onset of treatment (*P* > 0.1915, data not shown). Daily oral gavage of 18-month-old female mice with BAY for 8 weeks did not deleteriously affect liver or kidney function as shown by serum measurements of aspartate aminotransferase (AST), alanine aminotransferase (ALT), blood urea nitrogen (BUN), or creatine (Supplemental Figure 2, A–E). Although AhR activity has been reported as critical for maintenance of gut barrier function (24–27), orally delivered BAY treatment did not promote a leaky gut phenotype (Supplemental Figure 2F).

**Figure 2:**
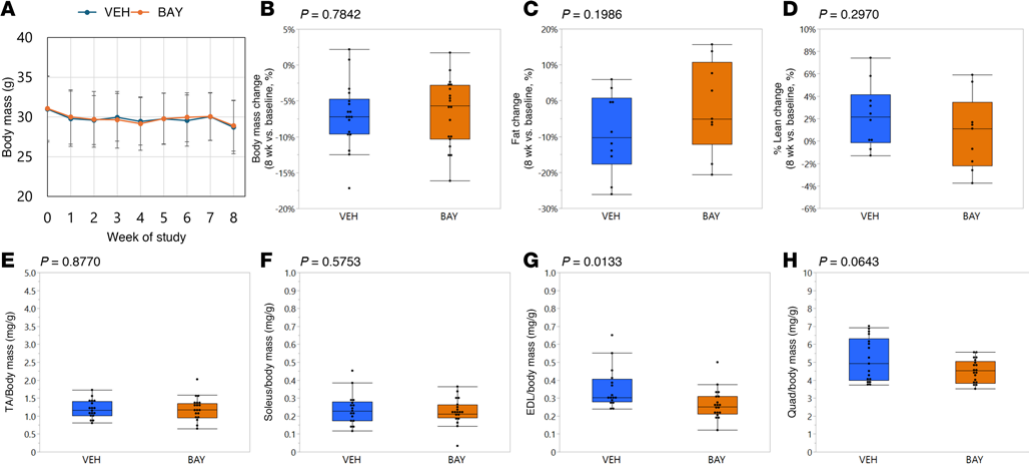
BAY treatment did not impact body mass but led to modest decreases in relative weights of fast-twitch muscles. Body mass was measured weekly (**A**), and showed no impact of BAY treatment over the course of the study (**B**). Fat mass (**C**) and lean mass (**D**) were measured prior to the onset of treatment and immediately prior to sacrifice and showed no impact of BAY treatment. Relative weights of muscles collected at sacrifice showed no effect on TA (**E**) or soleus (**F**) muscles, but BAY treatment reduced the relative weights of extensor digitorum longus (EDL) (**G**) and tended to reduce the weight of quadriceps (**H**) muscles as compared with VEH treatment. *P* values from Student’s *t* tests comparing groups are shown above each graph.

Longitudinal changes in body mass (Figure 2, A and B) and body composition (Figure 2, C and D) over the 8-week study were not different between VEH- and BAY-treated mice. Tissue weights at sacrifice showed no impact of BAY on muscle weights of the mixed fiber type TA or the predominantly slow-twitch soleus (Figure 2, E and F). Interestingly, BAY treatment promoted a relative decrease in weight of the fast-twitch extensor digitorum longus (EDL) and a mild trend for decreasing the weight of the quadriceps (Figure 2, G and H). Although the quadriceps is a mixed fiber type muscle like the TA, it is predominantly composed of fast-twitch (glycolytic) fibers, particularly in aged animals which were reported to show a decline in slow-twitch (oxidative) fiber content as compared with younger mice (28).

### BAY treatment prevented loss of muscle function during aging.

**Figure 3:**
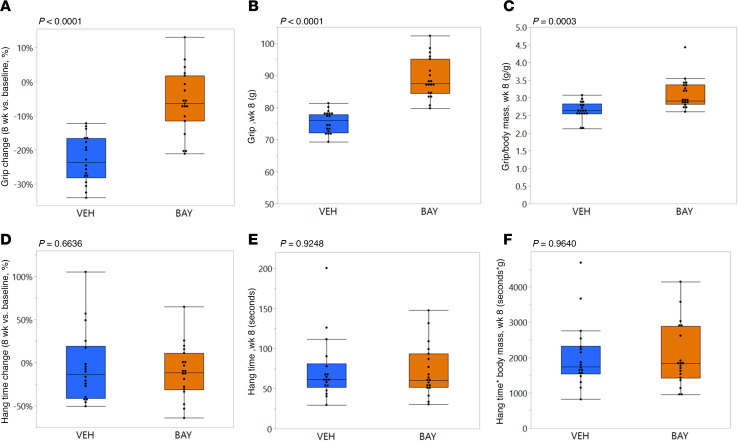
BAY treatment prevented a decline in forelimb grip strength over time. Grip strength (**A**–**C**) was measured prior to the onset of treatment and immediately prior to sacrifice. Longitudinal changes in grip strength for each mouse (**A**), as well as cross-sectional measurements of grip strength (**B**) and grip strength normalized to body mass (**C**) at 8 weeks of treatment were all significantly improved by BAY treatment. In contrast, neither longitudinal changes in hang time for each mouse (**D**), nor cross-sectional measurements of hang time (**E**) or hang time normalized to body mass (**F**) at 8 weeks of treatment were affected by BAY treatment.

Neither grip strength (*P* = 0.2858, data not shown) nor grip strength normalized to body mass (*P* = 0.3391, data not shown) were different between groups prior to the onset of treatment. The VEH-treated animals demonstrated a decline in forelimb grip strength over the course of the study, represented as a negative percentage change in grip strength between 0 and 8 weeks of treatment (Figure 3A), but this decline was prevented by BAY (*P* < 0.0001; Figure 3A). BAY-treated mice had greater grip strength (*P* < 0.0001; Figure 3B) and grip strength normalized to body mass (*P* = 0.0003; Figure 3C) as compared with VEH-treated mice after 8 weeks of treatment. In contrast, hang time testing of muscle endurance, which was also not different between groups at baseline (*P* > 0.9281; data not shown) did not change over the course of study (*P* = 0.6636; Figure 3D) and was not different between groups after 8 weeks of treatment (*P* > 0.9248; Figure 3, E and F).

### BAY treatment improved the integrity of the neuromuscular junction.

**Figure 4:**
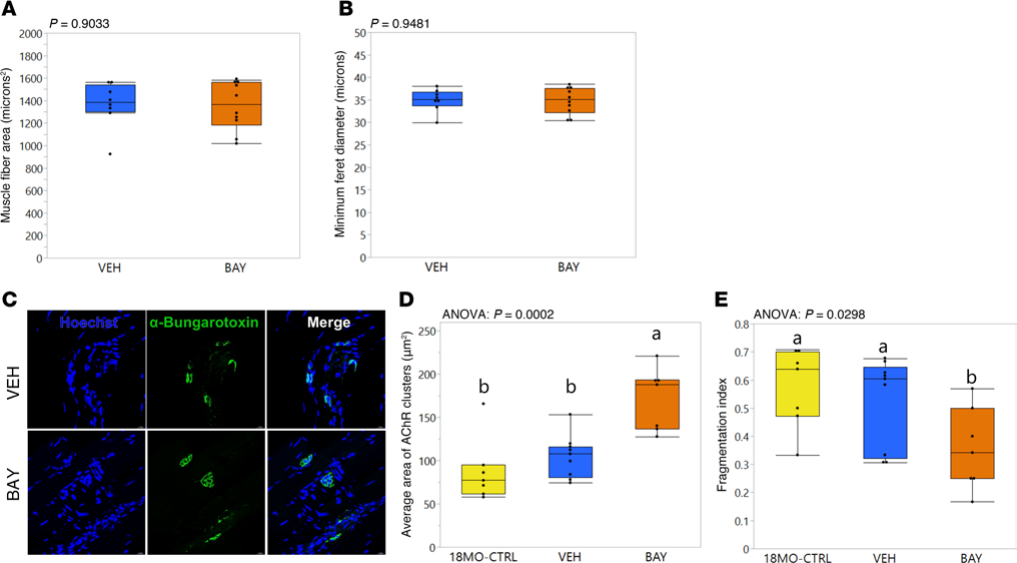
BAY treatment improved the integrity of the neuromuscular junction in skeletal muscle. Neither average muscle fiber cross-sectional area (**A**) nor minimum Feret diameter (**B**) in the TA muscle was affected by BAY treatment. Representative images of histological analyses of the neuromuscular junction (NMJ) via α-bungarotoxin staining (**C**) show superior integrity of the NMJ in BAY-treated mice, original magnification, ×40. The average area of acetylcholine receptors (**D**) and fragmentation index (**E**) of BAY-treated mice were superior compared with VEH-treated mice and with 18-month-old control animals. *P* values from Student’s *t* test (**A** and **B**) or 1-way ANOVA with Fisher’s LSD post hoc analysis (**D** and **E**) comparing groups are shown above each graph. Groups with different lower-case letters are significantly (*P* < 0.05) different from one another as shown by post hoc testing.

Surprisingly, despite the significant retention in grip strength observed in the BAY-treated mice, there were no differences between groups in muscle fiber average size (*P* = 0.9033; Figure 4A) or minimum Feret diameter (*P* = 0.9481; Figure 4B) in the TA. We therefore tested other potential contributing factors to muscle strength. Visualization of neuromuscular junctions (NMJs) in the TA revealed highly fragmented NMJs in the skeletal muscle of VEH-treated mice, whereas BAY-treated mice demonstrated excellent NMJ integrity (Figure 4C). BAY-treated mice also demonstrated a larger average area of acetylcholine receptor (AChR) cluster staining as compared with VEH-treated mice (*P*_posthoc_ = 0.0006) (Figure 4D) as well as lower fragmentation index values (*P*_posthoc_ = 0.0356) (Figure 4E). To determine whether this effect of BAY was due to preservation of the NMJ or rescue, we sacrificed an additional cohort of C57BL/6 females obtained from the National Institute on Aging (NIA) colony at 18 months of age, i.e., the age at which VEH or BAY treatments began. Surprisingly, NMJs in the TA of these 18-month-old control mice were indistinguishable from the 20-month-old VEH-treated mice in terms of average AChR cluster area and fragmentation index (Figure 4, D and E); *P*_posthoc_ = 0.3151 and *P*_posthoc_ = 0.5187, respectively), suggesting that the improvement of NMJ integrity in BAY-treated mice represented a “rescue” rather than “preservation” effect of the drug.

### BAY treatment demonstrated a mild beneficial effect on cortical bone.

**Figure 5:**
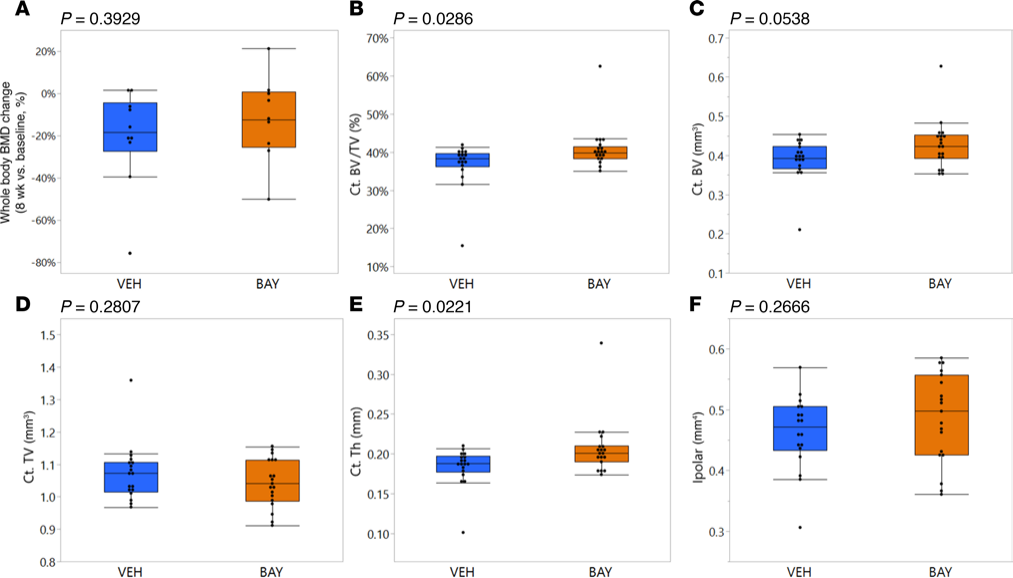
Cortical bone mass was mildly improved by BAY treatment. DXA scans were collected at baseline (18 months of age) prior to the onset of treatment and immediately prior to sacrifice (20 months of age). Longitudinal analyses of whole-body bone mineral density (BMD) showed no impact of BAY treatment (**A**). However, microCT analyses of the femur mid-diaphysis showed selective improvement in metrics of cortical bone mass in BAY-treated mice. (**B**) Cortical bone volume fraction, (**C**) cortical bone volume, (**D**) cortical bone tissue volume, (**E**) cortical thickness, and (**F**) polar moment of inertia. *P* values from Student’s *t* test comparing groups are shown above each graph.

Longitudinal changes in whole body bone mineral density (BMD) (*P* = 0.3929; Figure 5A), femur BMD (*P* = 0.7649; data not shown), and lumbar vertebrae BMD (*P* = 0.2790; data not shown) were not different between VEH- and BAY-treated groups. However, higher-resolution micro-computed tomography (microCT) measurements of cortical bone in the midshaft of the femur demonstrated that BAY-treated mice had a subtle but significantly greater cortical bone volume fraction (*P* = 0.0286; Figure 5B) as compared with VEH-treated mice, resulting from a strong trend for greater cortical bone volume (*P* = 0.0583; Figure 5C) without an impact on cortical bone tissue volume (*P* = 0.2807; Figure 5D). Cortical bone thickness was also significantly greater in BAY-treated as compared with VEH-treated mice (*P* = 0.0221; Figure 5E). Polar moment of inertia was not different between groups (*P* = 0.2666; Figure 5F). MicroCT measurements of cortical bone in the distal femoral metaphysis demonstrated that BAY-treated mice also showed modest but significant improvements in this site. While the BAY-treated mice did not show differences in cortical bone volume fraction (*P* = 0.1835; Supplemental Figure 3A) in the distal femur, they did develop greater cortical bone volume (*P* = 0.0217; Supplemental Figure 3B), cortical tissue volume (*P* = 0.0204; Supplemental Figure 3C), and cortical bone thickness (*P* = 0.0205; Supplemental Figure 3D), while also showing a mild trend for increased polar moment of inertia (*P* = 0.0950; Supplemental Figure 3E). Cortical bone porosity was not different between groups (*P* = 0.1609; Supplemental Figure 3F).

We conducted dynamic histomorphometry in the femur mid-diaphysis to determine whether periosteal or endocortical bone formation rates were affected by BAY treatment, but failed to detect any impact of BAY treatment on these metrics (Supplemental Table 1), although periosteal double labeling was largely absent in these aged VEH- and BAY-treated mice, precluding accurate quantification of periosteal bone formation metrics. Serum markers of bone resorption activity (TRAcP5b) and bone formation activity (P1NP) were not different between groups (*P* > 0.2011; Supplemental Figure 4, A and B). Likewise, neither histological metrics of bone marrow adiposity (*P* > 0.2283, Supplemental Figure 4, C and D) nor inflammatory cytokines in the serum or bone marrow fluid (Supplemental Table 2) were affected by BAY treatment. Other bone architectural properties, including trabecular bone properties in the distal femoral metaphysis and lumbar vertebrae, were not different between groups (*P* > 0.3660; Supplemental Table 3).

### Primary BMSCs from BAY-treated mice produced more colonies in vitro, resulting in increased formation of mineralized matrix.

**Figure 6:**
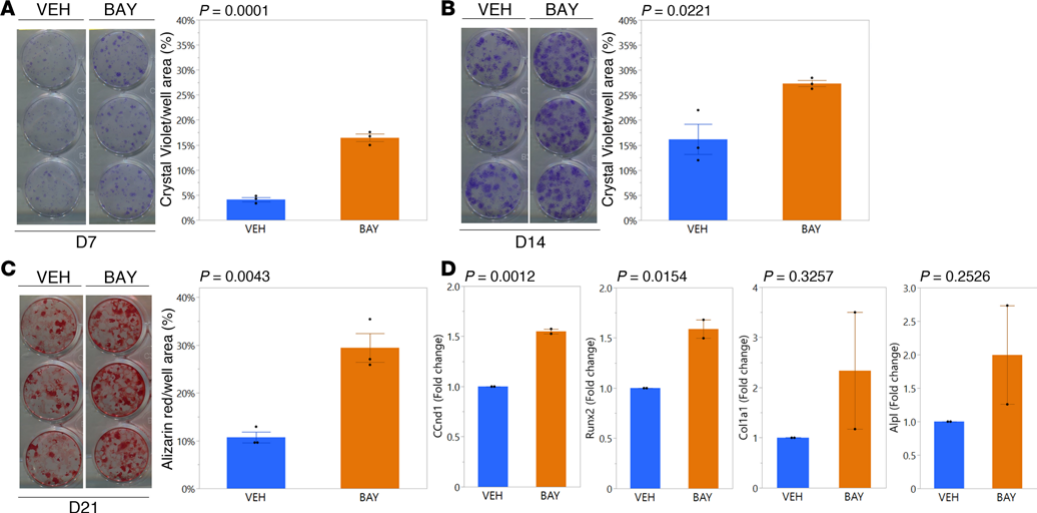
BMSCs from BAY-treated mice showed enhanced colony formation and produce more matrix in vitro. Crystal violet staining of BMSC-derived colonies on (**A**) day 7 and (**B**) day 14 in culture show increased colony formation by cells isolated from BAY-treated mice as shown by quantification of crystal violet–stained area normalized to total well area. (**C**) Alizarin red staining (normalized to total well area) was also enhanced in BMSC-derived osteoblast cultures isolated from BAY- as compared with VEH-treated mice. Each well shown represents 1 technical replicate culture. (**D**) Gene expression analyses from BMSC-derived osteoblast cultures on day 21 in culture. *P* values from Student’s *t* tests comparing groups are shown above each graph.

Consistent with the observed increase in cortical bone mass, primary BMSCs isolated from BAY-treated mice showed enhanced production of mineralized matrix via alizarin red staining after 21 days in osteogenic culture (Supplemental Figure 5). To understand this effect, we conducted colony-forming unit–fibroblast (CFU-F; BMSC culture medium) and colony-forming unit–osteoblast (CFU-Ob; osteogenic culture medium) assays with primary BMSCs from a second cohort of animals. Primary BMSCs isolated from the BAY-treated mice demonstrated a propensity for enhanced proliferation, as colony formation visualized by crystal violet staining was significantly greater in cultures derived from the BAY-treated as compared with VEH-treated mice at both day 7 (Figure 6A) and day 14 (Figure 6B) of culture, resulting in greater mineralized matrix formation by day 21 in culture (Figure 6C). Consistent with staining results, mRNA expression from CFU-Ob collected at day 21 in culture demonstrated increased expression of cyclin D1 (Ccnd1), suggesting increased proliferation, and increased expression of Runx2, suggesting increased commitment to the osteoblast lineage, in the cultures derived from the BAY-treated as compared with VEH-treated mice (Figure 6D). However, expression levels of more mature osteoblastic markers like alkaline phosphatase (Alpl) and type 1 collagen (Col1a1) were not different between groups (Figure 6D).

### CH-223191 treatment demonstrated a mild beneficial effect on aged bone and muscle.

**Figure 7:**
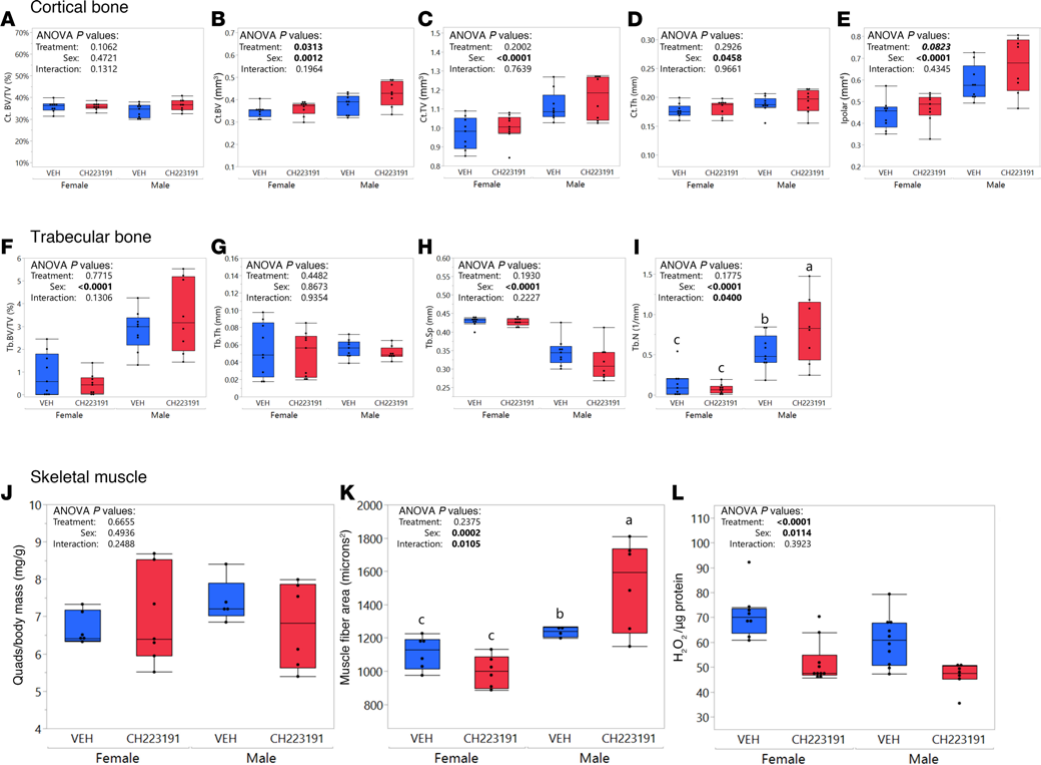
CH-223191 treatment is beneficial for cortical bone and skeletal muscle. (**A**–**E**) Cortical bone mass, as measured by microCT; cortical bone volume fraction (**A**), cortical bone tissue volume (**C**), and cortical bone thickness (**D**) were not affected by CH-223191 treatment, while cortical bone volume (**B**) was significantly greater in CH-223191– as compared with VEH-treated mice, and similar trends are shown for polar moment of inertia (**E**). Trabecular bone (**F**–**I**) was largely unaffected by CH-223191 except for a demonstration of increased trabecular number in male but not female CH-223191–treated mice (**I**). While quadriceps mass was not affected by CH-223191 (**J**), average muscle fiber size in the TA muscle was increased in male CH-223191–treated mice (**K**), and metrics of oxidative stress (from Amplex Red assays) were reduced by CH-223191 in skeletal muscle from both sexes (**L**). *P* values from 2-way ANOVA with Fisher’s LSD post hoc analysis comparing groups are shown on each graph; group with different superscript letters are significantly (*P* < 0.05) different from one another as shown by post hoc testing.

As BAY showed beneficial effects in both skeletal muscle and bone, we sought to determine whether this result could be replicated with another AhR antagonist. CH-223191, a well-known AhR antagonist (20), was administered to male and female C57BL/6 mice beginning at 16 months for a period of 12 weeks. Mice treated with CH-223191 had significantly (*P*_treatment_ = 0.0313) greater cortical bone volume than VEH-treated controls, although cortical bone volume fraction and cortical bone tissue volume were unaffected by treatment (Figure 7, A–C). While cortical bone thickness was not affected by treatment (*P*_treatment_ = 0.2926; Figure 7D), mice treated with CH-223191 showed a trend for increased polar moment of inertia (*P*_treatment_ = 0.0823; Figure 7E). These results suggest that CH-223191, like BAY, shows a mild, beneficial effect on cortical bone in aged mice. In the trabecular bone of the distal femoral metaphysis, CH-223191 treatment did not affect trabecular bone volume fraction, trabecular thickness, or trabecular separation (*P*_treatment_ > 0.1930; Figure 7, F–H) but showed a sexually dimorphic benefit for trabecular number, with increased trabecular number in CH-223191–treated male mice as compared with VEH-treated controls and no effect in female mice (Figure 7I). With regard to skeletal muscle, although quadriceps mass was not significantly affected by CH-223191 treatment (Figure 7J), TA fiber size was selectively increased by CH-223191 in males (Figure 7K), and TA from CH-223191-treated mice demonstrated a significant reduction in oxidative stress (as shown by H_2_O_2_ concentration in Amplex Red assays, Figure 7L; one male VEH-treated sample was excluded from analyses, as it was identified as a statistical outlier by Grubbs test) (29). Together, these data suggest that pharmacologically inhibiting AhR with CH-223191, as with BAY, has beneficial effects on both bone and muscle.

### RNA-seq and proteomics reveal cellular metabolic pathways affected by BAY in musculoskeletal tissues.

**Figure 8:**
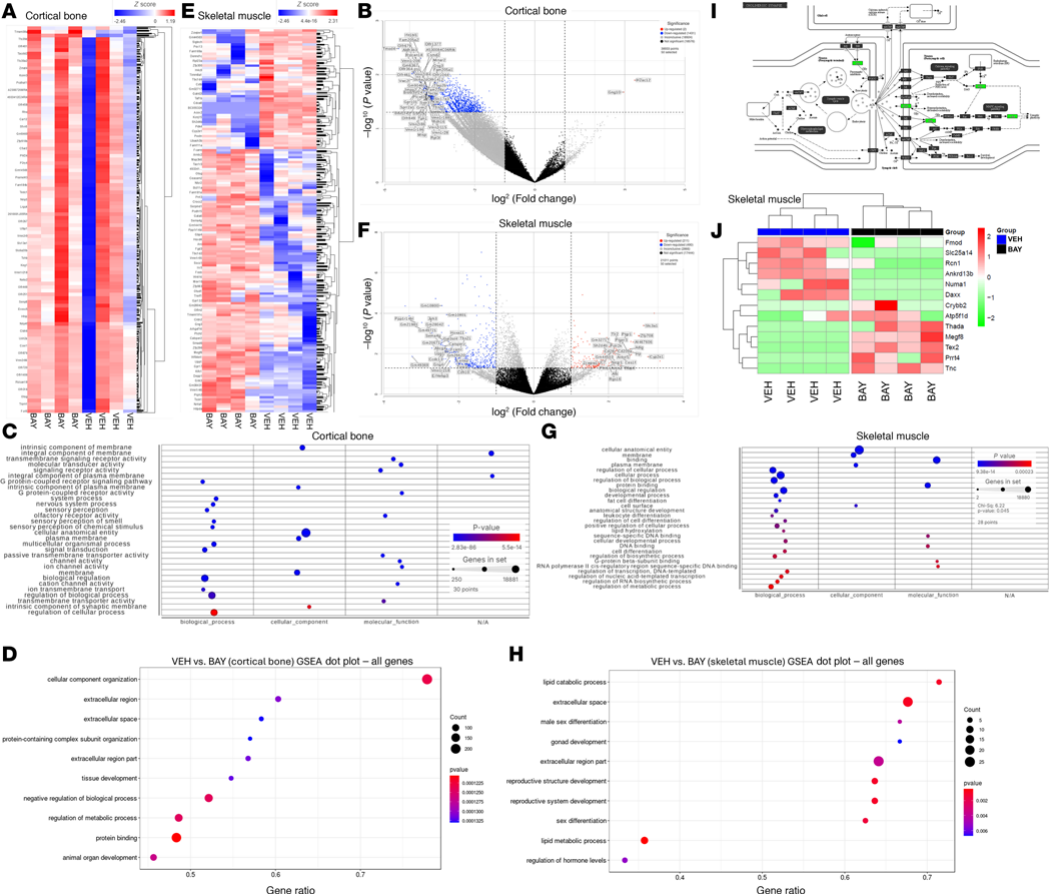
Transcriptomic and proteomic data supported the histological data and suggested that BAY preserves skeletal muscle function in part by maintaining molecular mediators of the NMJ in aged mice. Heatmaps (**A** and **E**), volcano plots (**B** and **F**), Gene Ontology plots of protein coding genes and biological processes (**C** and **G**), and GSEA plots (**D** and **H**) and are shown. Genes found in the cholinergic synapse pathway (**I**) were expressed at higher levels in the BAY-treated as compared with VEH-treated mice. (**J**) Proteomics analyses of skeletal muscle lysates identified differentially expressed proteins between BAY- and VEH-treated mice.

In bulk RNA-sequencing (RNA-seq) analyses comparing the effects of BAY versus VEH treatment in cortical bone, of a total of 38,933 genes, 1433 (847 protein coding) were differentially expressed between groups, with a *P* value of less than 0.05 and an absolute fold change of 2 or more (Figure 8, A and B). Gene Ontology analyses revealed 10 pathways differentially regulated between the 2 groups, with 7 additional pathways that approached significance (Supplemental Table 4 and Figure 8C); “Circadian Entrainment” represented the highest enrichment score. Gene set enrichment analysis (GSEA) of protein-coding genes demonstrated that “cellular component organization” was significantly altered by BAY treatment, along with “protein binding” and “animal organ development” appearing in the top 10 results (Figure 8D).

For the comparison of skeletal muscle between BAY- and VEH-treated mice, of a total of 21,011 genes, 701 (327 protein coding) were differentially expressed, with a *P* value of less than 0.05 and an absolute fold change of 2 or more (Figure 8, E and F). The AhR target gene, Cyp1a1, was suppressed in BAY- versus VEH-treated skeletal muscle (–2.32-fold, *P* = 0.0217), validating earlier analyses with RNAscope (Figure 1). Gene Ontology analyses revealed 30 pathways differentially regulated (*P* < 0.05, enrichment score > 3.0) between groups, with “Circadian entrainment” representing the largest enrichment score (Supplemental Table 5 and Figure 8G). GSEA of protein-coding genes demonstrated that “lipid catabolic processes” were significantly altered by BAY treatment, along with “lipid metabolic processes” and “regulation of hormone levels” appearing in the top 10 results (Figure 8H). Genes such as Fos, Gng3, Gng4, and Prkcg found in the cholinergic synapse pathway (Figure 8I) were expressed at higher levels in the skeletal muscle of BAY-treated as compared to VEH-treated mice. Consistent with this finding, proteomics analyses revealed that proteins including Megf8 (multiple epidermal growth factor-like domains 8) and Tnc (tenascin-C) were expressed at higher levels in BAY-treated as compared with VEH-treated mice (Figure 8J); pathway analyses demonstrated that Megf8 and Tnc are related to biological processes including neuron projection fasciculation, axonal fasciculation, neuron recognition, and axon regeneration at NMJs. Taken together, the transcriptomic and proteomic data support the histological data and suggest that BAY preserves skeletal muscle function in part by maintaining molecular mediators of the NMJ in aged mice.

### Positive effects of AhR inhibition on NMJ and fiber size can be recapitulated in C2C12 cells.

**Figure 9:**
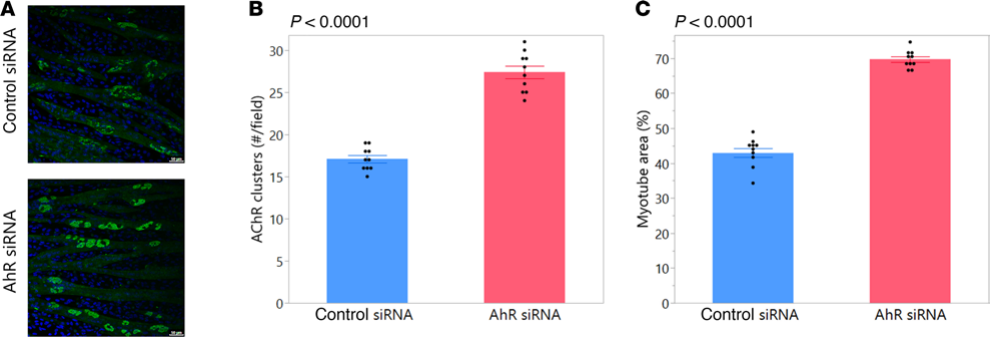
AhR knockdown by siRNA is beneficial to myofibers. (**A** and **B**) C2C12 cells treated with siRNA against AhR (AhR siRNA) demonstrated an increased number of acetylcholine receptor (AChR) clusters as compared with cultures treated with a nontargeting control siRNA. Representative images of α-bungarotoxin staining are shown in **A**. Scale bars: 50 μm. (**C**) C2C12 cells treated with siRNA against AhR and subjected to myogenic culture demonstrated an increase in myotube area as compared with control siRNA–treated cells. Group means ± SEM are shown; *P* values for Student’s *t* tests comparing groups are shown above each graph.

Results from the in vivo studies with BAY and CH-223191 suggested that pharmacological inhibition of AhR is beneficial for myofibers. To determine whether this occurs in a direct, cell-autonomous fashion, we subjected C2C12 myoblasts to myogenic culture conditions in the presence of control siRNA or siRNA targeting AhR. Similar to in vivo studies with BAY, AhR siRNA promoted an increase in the number of AChR clusters in C2C12 cells (Figure 9, A and B). This was associated with an increased myotube area in AhR siRNA as compared with control siRNA culture conditions (Figure 9C). Taken together, these results suggest that AhR inhibition has a direct, beneficial effect on myofibers that promotes improvements in skeletal muscle mass.

## Discussion

As life expectancy increases on a global scale (30), new strategies are needed to combat musculoskeletal decline associated with impaired mobility and poor quality of life (31–33). While drug therapies, including bisphosphonates, anti-RANKL antibodies (denosumab), anti-sclerostin antibodies (romosozumab), and teriparatide, already exist to mitigate aging-related bone loss to some degree, a dearth of pharmacological therapeutic options is currently available to address issues related to dynapenia and sarcopenia with age (34, 35). Data presented here suggest that pharmacologically inhibiting AhR may represent a strategy to abrogate age-related declines in both cortical bone mass and muscle function. As AhR antagonists have shown positive outcomes in clinical trials as anticancer therapeutics (3, 21), this suggests a potential opportunity for eventual drug repurposing to address musculoskeletal decline associated with aging, although further translational studies will be needed to more firmly establish this premise.

The 2 AhR antagonists tested here, BAY and CH-223191, showed similar but not identical effects in bone and muscle. As one example, while grip strength was preserved in BAY-treated as compared with VEH-treated mice, no changes were seen in hindlimb muscle fiber size. In contrast, male CH-223191–treated mice showed increases in average fiber size in TA muscles as compared with VEH-treated mice. Similarly, although BAY and CH-223191 both tended to increase cortical bone volume, cortical bone thickness was increased by BAY but not by CH-223191 (Figures 5 and 7). Differences between these 2 compounds may be due in part to differences in statistical power, as we had larger sample sizes (*n* > 15/group) for most endpoints in BAY studies as compared with CH-223191 studies. In addition, while BAY and CH-223191 both target AhR to antagonize its function, they are not identical in structure or mechanism of action. BAY, as first reported in 2023, was identified from a screen of 3.2 million compounds as capable of direct competitive binding to the AhR and inhibiting its activation by both exogenous and endogenous ligands (21). While CH-223191, like BAY, also competitively binds to AhR, it is more effective at inhibiting AhR activation by halogenated aromatic hydrocarbons like 2,3,7,8-tetrachlorodibenzo-*p*-dioxin (TCDD) as compared with other activators like polycyclic aromatic hydrocarbons or flavonoids including beta-naphthoflavone (20). CH-223191 promoted proliferation of certain cell populations in an AhR-independent fashion (36), whereas short-term culture with BAY had no effect on proliferation of PyMT murine mammary cancer cells, and longer-term culture (>6 weeks) with BAY reduced the proliferation of these cells in a manner similar to AhR genetic knockout (37). In the current study, primary BMSCs isolated from BAY-treated mice demonstrated enhanced proliferative activity that likely contributed to the increased mineralized matrix production by BMSC-derived osteoblasts (BMSC-OBs) from BAY-treated mice. Similarly, our previous studies demonstrated that murine and human mesenchymal stem cells show suppressed secretion of the pro-osteogenic factor stromal cell–derived factor 1 (also known as Cxcl12) when treated with the AhR ligand kynurenine, and this effect was prevented by CH-223191 (5). Regardless of their molecular differences, it is notable that both compounds showed evidence of a beneficial impact in aged musculoskeletal tissues, supportive of AhR representing a therapeutic target for aging-related frailty.

The administration of BAY demonstrated a mild, but significant, positive impact on cortical bone, although no effect was observed on trabecular bone. In the distal femoral metaphysis, this discrepancy could be attributed in part to low abundance of trabecular bone present in 18-month-old female mice at the onset of treatment, but lumbar vertebral trabecular bone also failed to show an impact of BAY. Interestingly, a recent publication (38) compared gene expression between cortical and trabecular bone via bulk RNA-seq, and in this dataset, both AhR and its target gene Cyp1b1 were more highly expressed in cortical bone than in trabecular bone (AhR: +2.31-fold in cortical vs. trabecular bone, *P* = 0.000136; Cyp1b1: +2.38-fold in cortical vs. trabecular bone, *P* = 7.43 × 10^–6^), as was expression of Slc7a5 (also known as LAT1), the proposed transporter responsible for import of tryptophan and its downstream metabolites like kynurenine (+2.50-fold in cortical vs. trabecular bone; *P* = 2.30 × 10^–14^; Supplemental Table 3 in ref. 38). Accordingly, it is possible that AhR-mediated signaling plays a more critical role in cortical than trabecular bone homeostasis during aging, but this will need to be functionally tested in future studies.

Consistent with the current studies, we (2, 3, 12, 15, 39) and others (18, 23, 40) have previously suggested an important role for AhR in mediating bone and muscle cell function. As one example, pharmacological and genetic blockade of AhR attenuated osteoclastogenic effects of kynurenine (2), consistent with the observation that both whole-body knockout and tissue-targeted knockout of AhR with cathepsin K–Cre promoted reduced bone resorption (18). A recent publication demonstrated that the AhR-activating ligand TCDD promoted craniofacial malformations, in part due to suppression of osteogenic genes in mesenchymal progenitor cells (23), and these antiosteogenic effects could be prevented in vitro via AhR inhibition with BAY (23). In muscle, targeted deletion of AhR with HSA-MCM-Cre preserved muscle mass and contractile function in a model of chronic kidney disease (41). While these previous studies establish a strong premise for the general importance of AhR-mediated signaling in bone and muscle, the current studies add insight into the relevance of this mechanism in the context of aging. As circulating and bone marrow niche levels of the AhR ligand kynurenine increase with age and are associated with musculoskeletal dysfunction (5–8, 10), it is possible that the benefits seen here with AhR inhibition reflect a mitigation of this kynurenine-mediated effect. However, although AhR inhibition with BAY and CH-223191 showed a benefit to aged skeletal muscle and the NMJ, a previous report demonstrated that AhR inhibition via CH-223191 was unable to prevent oxidative stress induced by in vitro kynurenine treatment in C2C12 cells (12). Similarly, whole-body AhR-knockout mice did not show an altered response to kynurenine treatment in vivo, although it is important to note that whole-body AhR-knockout mice exhibit a spectrum of systemic defects, including cardiovascular and gastrointestinal tract lesions (42) and an accelerated aging phenotype consisting of increased metrics of inflammation and cognitive dysfunction (43). A previous report suggests that functional benefits derived from targeted deletion of AhR in myofibers were linked to protection against indoxyl sulfate, a uremic metabolite derived from tryptophan (41). Future studies will be needed to determine which endogenous ligands (e.g., tryptophan) are responsible for activating AhR in the musculoskeletal system during aging to better understand the molecular mechanism by which AhR inhibition provides a functional benefit to aged bone and muscle tissue function.

It is important to note that AhR-mediated signaling has been reported to play a critical role in gut homeostasis where it protects against inflammation, maintains barrier integrity, and mediates immune responses (24–27, 44). Similarly, although AhR-activating tryptophan metabolites like kynurenine are generated via indoleamine 2,3-dioxygenase 1 (IDO1) in peripheral tissues such as the musculoskeletal system and tryptophan 2,3-dioxygenase (TDO) in the liver (3), these metabolites and others (like indoles) are also actively generated from tryptophan by the microbiome residing in the gastrointestinal tract (40). As the pharmacological AhR antagonists utilized in the current study were administered systemically, and in the case of BAY via oral delivery, at present we cannot confirm whether the musculoskeletal benefit of these drugs in aging models tested here represents a direct effect on musculoskeletal tissues or a downstream effect of altered signaling in other locations such as the gut. However, previous studies demonstrate that loss of AhR-mediated signaling in the gut often carries negative consequences. As one example, conditional deletion of AhR in intestinal epithelial cells promoted increased susceptibility to infection (45). Similarly, AhR expression is downregulated in inflammatory bowel conditions, including Crohn disease (46), whereas AhR activation is associated with inhibition of intestinal inflammatory responses (46, 47). Thus, while we cannot rule out an impact on the gut, the fact that AhR inhibition did not alter local or systemic indicators of inflammation or gut barrier integrity suggests that the improved musculoskeletal function observed with AhR inhibition in the current study is unlikely to be an indirect effect of gut-mediated changes.

There are several limitations to our study that warrant consideration. For example, it is important to note that despite its prevention of age-related reductions in grip strength, BAY treatment tended to decrease the weights of the EDL and quadriceps muscles, but not the soleus or TA muscles, suggesting an as-yet unexplained reduction in the mass of muscles predominantly composed of fast-twitch fibers that could be physiologically detrimental. Muscle function was assessed via forelimb grip strength testing, whereas these declines in muscle mass were observed in the hindlimb, so at present the 2 observations cannot be functionally linked. Future studies aimed at directly assessing muscle function in the hindlimb, as well as more in-depth analyses such as fiber typing, will be needed to fully understand the potential benefits and risks of BAY treatment in terms of muscle physiology. Similarly, our mechanistic insights into how BAY promoted positive effects in cortical bone are limited, as although we detected increased matrix production by BMSC-OBs from the BAY-treated mice in vitro and biological pathways enriched by BAY treatment in RNA-seq analyses, we acknowledge the limitation that our in vitro sample sizes for gene expression analyses in the BMSC-OB cultures were small (*n* = 2 biological replicates per group), and our variability in gene expression was high across the cortical bone samples obtained from VEH-treated mice for RNA-seq analyses. We were unfortunately unable to add primary osteoclast cultures to our analyses, which despite the lack of change in Tracp5b serum levels would have been beneficial for evaluating potential osteoclast-related contributions to the cortical bone phenotypes observed. Our RNA-seq and proteomics datasets were also limited by a small sample size (*n* = 4 mice per group), necessitating less stringent thresholds for statistical definitions of differential expression to permit exploratory pathway analyses. Future studies will be needed to elucidate the molecular mechanisms by which BAY is beneficial for cortical bone and skeletal muscle in aged female mice.

In conclusion, pharmacological inhibition of AhR improved cortical bone mass and skeletal muscle function in aged mice, an effect that is attributed at least in part to improvement in the integrity of the NMJ. These data support AhR as a therapeutic target for improving musculoskeletal health during aging.

## Methods

Further information can be found in Supplemental Methods.

### Sex as a biological variable.

Male and female C57BL/6 mice were used in preliminary studies (16-week-old mice) with BAY (Supplemental Methods), but subsequent studies on aged mice focused on females, as female mice showed stronger responses to BAY treatment than males (Supplemental Figure 1). Both male and female C57BL/6 mice were used in studies with CH-223191.

### In vitro studies: BAY as an AhR antagonist.

Immortalized ST2 cells (ATCC) (48) were seeded in 12-well plates at 20,000 cells/well. Beginning 24 hours after seeding, the cells were transiently transfected with 500 ng of an AhR reporter plasmid (XRE-binding sites driving expression of firefly luciferase) (49) or Renilla luciferase plasmid (RLnull; 10 ng) using Lipofectamine 3000 (Thermo Fisher Scientific, L3000015), as described previously (15). After 24 hours, cells were treated with 1 μM BAY (Targetmol, T10270) or its VEH (DMSO) for 3 hours, after which the media were changed again to expose the cells to kynurenine (10 μM; MilliporeSigma, K8625) in the presence of BAY or VEH for an additional 24 hours. AhR transcriptional activity was quantified with the Promega Dual-Luciferase Reporter Assay System (Promega Corporation, E1960) with luminescence measured using a BioTek Cytation 5 multimode microplate reader (Agilent). Firefly luciferase values from 3 technical replicates were normalized to Renilla luciferase values, and fold change in transcriptional activity was calculated relative to the VEH-treated control.

### In vitro studies: AhR knockdown by siRNA.

C2C12 cells (ATCC) were seeded at 100 cells/mm² and cultured in DMEM with 1% penicillin/streptomycin. One day after seeding, medium was switched to DMEM plus 1% penicillin/streptomycin supplemented with 2% horse serum to stimulate myogenic differentiation. To induce AChR cluster formation, dishes were coated with recombinant laminin 121 (Biolamina, LN121-0501) (50). Cells were monitored after 48 hours in culture and transfection performed on 75%–80% confluent cells. Media were replaced, 750 μL of Opti-MEM (Gibco, 31-985-070) was added to each well, and 3 μl of 10 μM control (Silencer Select Negative Control No. 1 siRNA; Thermo Fisher Scientific, 4390843) or AhR (Silencer Select AhR siRNA; Thermo Fisher Scientific, 4390771) was transfected using Lipofectamine RNAiMAX (Invitrogen, 13778075). Cells were cultured in the presence of horse serum for 96 hours, fixed with 4% paraformaldehyde for 7 minutes, washed with 1× PBS 3 times, blocked in blocking buffer (2% bovine serum albumin, 0.2% normal goat serum, and 0.05% Triton X-100) for 30 minutes, and incubated for 3 hours or overnight with Alexa Fluor 488–α-bungarotoxin (1 ng/μL; Invitrogen, B13422). Cells were washed 3 times with 1× PBS, slides mounted with DAPI mounting medium, sealed with a coverslip, and imaged using confocal microscopy (Leica STELLARIS). AChR clusters were measured as total number of stained clusters per field. Myotube size was measured as total myotube area per field for approximately 5 images per treatment group.

### In vivo studies: BAY treatment.

Female C57BL/6 mice (*n* = 40) were obtained from the NIA colony. Mice were group-housed in an accredited facility and maintained on a 12-hour light/dark cycle. Mice were provided food (Teklad 2018, Envigo) and water ad libitum, and randomly allocated to receive VEH (ethanol/Solutol/water, 10:40:50 by volume) or BAY (30 mg/kg body mass; Targetmol, T10270 suspended in VEH) via daily oral gavage 5 days/week for 8 consecutive weeks (*n* = 19–20/group). Body mass was measured weekly. One mouse died prior to beginning treatments, one mouse died between weeks 4 and 5 of treatment (VEH group) from unknown causes, and one mouse was discovered to have a large liver tumor at sacrifice and was excluded from further study (BAY group), resulting in final sample sizes of VEH: *n* = 18, and BAY: *n* = 19. All mice received intraperitoneal injections of 10 mg/kg calcein (Sigma-Aldrich, C0875) at 8 days and 1 day prior to being euthanized via carbon dioxide inhalation. Blood collected via cardiac puncture at sacrifice was allowed to coagulate, and serum was separated by centrifugation (BD Microtainer, 365967) and stored at –80°C until analysis. Skeletal muscles (TA, EDL, soleus, quadriceps), long bones, and vertebrae were collected at sacrifice.

### In vivo studies: CH-223191 treatment.

Male and female C57BL/6 mice (16 months old) were obtained from the NIA rodent colony. Mice were treated with VEH (DMSO) or CH-223191 (3.3 mg/kg body mass; Cayman Chemical, 16154) via daily intraperitoneal injection 3 days/week for 12 consecutive weeks (*n* = 6/group). TA, quadriceps, and long bones were collected at sacrifice.

### Gut barrier function.

As AhR has been reported to maintain gut barrier function (24–27), gut permeability was assessed via FITC-dextran (51, 52). Mice were gavaged with 100 μL FITC-dextran (100 mg/mL; 10 kDa) after overnight fast. Blood was collected from facial veins 90 minutes after gavage. Serum was analyzed by fluorimetry to determine the FITC-dextran concentration.

### Skeletal muscle function.

Muscle function was assessed at baseline (immediately prior to treatment) and after 8 weeks of treatment. Muscle endurance was quantified via wire hang-time testing, where hang time (in seconds) was measured. Forelimb grip strength (in grams) was assessed with a grip strength meter (Bioseb, BIO-GS3). Hang time and grip strength were normalized to body mass to account for any differences in relative muscle sizes between mice.

### Body composition and BMD.

Mice (*n* = 10 VEH; *n* = 9 BAY) were anesthetized using isoflurane and subjected to dual-energy x-ray absorptiometry (DXA) scans using a commercial system (Kubtec PARAMETER 3D cabinet, KUB Technologies). BMD and body composition were analyzed using the manufacturer’s software (Kubtec Digimus) for the whole body (excluding head) and regions of interest including the femur midshaft (middle third of diaphysis) and spine (L3–L5 vertebrae). Scans were collected at baseline (before the start of treatment) and after 8 weeks of treatment and analyzed as percentage change in each endpoint as compared to baseline.

### RNAscope analysis of AhR and Cyp1a1 mRNA.

Tibias and quadriceps from BAY- and VEH-treated mice (*n* = 3/group) were prepared for cryosectioning and RNAscope analyses, as described previously (15). Tissues were dehydrated in sucrose suspended in 0.5% formaldehyde, embedded in SCEM compound (Section Lab-Co, C-EM001), and cryosectioned (10 μm thickness) using the Kawamoto cryofilm method [Section Lab-Co, 3C(16UF) 2.0 cm] (53). Sections were immersed in PBS for 5 minutes to remove embedding compound and baked in a 60°C oven for 30 minutes followed by immersion in 10% neutral buffered formalin at 4°C for 15 minutes. Sections were moved through a graded ethanol series (50% to 100%) and air dried for 5 minutes, after which AhR and Cyp1a1 mRNAs were probed following manufacturer protocols (ACDBio). Sections were incubated with hydrogen peroxide solution for 10 minutes at room temperature and digested with protease III reagents for 45 minutes in a 40°C oven. Probes were added to sections at 1:50 dilution, incubated for 2 hours in a 40°C hybridization oven, and incubated with 5× saline-sodium citrate (SSC) (Fisher BioReagents, BP1325-1) buffer overnight. The next day, the RNAscope Multiplex Fluorescent Detection Reagents v2 kit was used to amplify probe signal, followed by incubation with horseradish peroxidase (HRP) for 15 minutes and a 30-minute incubation with 570 nm Opal dye (Akoyo Biosciences, FP1488001KT) and 620 nm Opal dye (Akoyo Biosciences, FP1495001KT) at a dilution of 1:1000. Finally, an HRP blocker was added to the sections for 15 minutes and the nuclei were stained with Hoechst dye. Two *Z*-stack images were taken per section using a Leica STELLARIS confocal microscope.

### Inflammatory cytokines, liver, and kidney function.

Inflammatory cytokines were measured in serum and bone marrow fluid using a Legendplex panel (Mouse Inflammation 13-Plex). Metrics of liver function (AST, ALT, AST/ALT ratio) and kidney function (BUN, creatinine) were measured in the serum from a subset of mice (*n* = 6 VEH; *n* = 6 BAY) with commercial assays (Mouse ALT ELISA Kit, Abcam, ab282882; Mouse AST ELISA Kit, Abcam, ab263882; Mouse Creatinine Assay Kit, Crystal Chem, 80350; and Invitrogen Urea Nitrogen Colorimetric Detection Kit, EIABUN).

### Skeletal muscle histomorphometry and NMJ histology.

TA muscles were histologically prepared. Metrics of muscle fiber size (minimum fiber Feret diameter and average fiber cross-sectional area) were quantified from trichrome- or H&E-stained sections. The integrity of NMJs was assessed via α-bungarotoxin staining. TA muscles were fixed in 10% formalin for 2 hours followed by 30% sucrose in 1× PBS for 24 hours at 4°C. Muscles were cryosectioned (30 μm) in parallel with muscle fiber orientation, permeabilized with 0.3% Triton X-100 in 1× PBS, and stained with 1 μg/mL α-bungarotoxin (Biotium, 00005-100μg) in 1× PBS in a dark humidified chamber. Sections were counterstained with 0.005 mg/mL Hoechst (Invitrogen, H1399) in 1× PBS prior to mounting (Vectamount, Vector Laboratories, H-5501) and imaged on a Leica STELLARIS confocal microscope using a 40× oil immersion objective. Average area of AChR clusters and fragmentation index were quantified with ImageJ as described previously (54). Fragmentation index, which reflects NMJ structural integrity and arrangement of synaptic components, was calculated as 1 – (1/N AChR clusters), where values closer to zero represented solid, plaque-like endplates (i.e., low fragmentation) and values closer to 1 represented dispersed structures (i.e., high fragmentation).

### Amplex Red assay.

TA muscles were utilized for measurement of hydrogen peroxide concentration using Amplex Red assays (Thermo Fisher Scientific, A22188) as described previously (55). Amplex Red reagent (100 μM) and 0.2 U/mL HRP prepared in reaction buffer was added (50 μL) to TA samples, incubated for 30 minutes at room temperature in the dark, and fluorescence measured at excitation/emission of 550 nm/590 nm on a BioTek Synergy H1 multi-mode reader. Protein concentration (Bio-Rad Protein Assay) was used to normalize hydrogen peroxide content as described previously (55).

### MicroCT.

Femurs and lumbar vertebrae were scanned in a SkyScan 1272 microCT instrument (Bruker MicroCT). Each sample was stabilized in cotton gauze in microcentrifuge tubes filled with 70% ethanol. Projection images were acquired using an image pixel size of 9.5–11 μm^2^ at a camera resolution of 1224 × 820 pixels. The x-ray source was set to a voltage of 70 kVp at 142 μA. A 0.5 mm aluminum filter was used for beam-hardening correction. Each specimen was rotated 180° in 0.5° steps, with 4 averaged frames acquired per step using an integration time of 620 ms. A random movement setting of 15 was applied. Three hundred eighty-six projections were reconstructed into cross-sectional images using NRecon software (Bruker, v1.7.4.6). A software beam-hardening correction of 45% and a ring artifact reduction of 4 was applied across all specimens. Reconstructions were loaded into CTAn software (Bruker) for morphometry. Three representative volumes of interest (VOIs) were defined in each femur and 1 VOI was defined in each L4 vertebra. Femoral trabecular bone and metaphyseal cortical bone were studied in the distal femoral metaphysis, in a region of interest 100 slices in length beginning immediately above the primary spongiosa. Femoral diaphyseal cortical bone was studied in the mid-diaphysis, in a region of interest 50 slices in length, beginning 500 slices (scaled to bone length) proximal to the distal femoral metaphysis growth plate. Trabecular bone in the L4 vertebra was measured in the midpoint of the L4 vertebral body in a region of interest (ROI) that was 25 slices in length. Contouring for trabecular VOIs was performed manually, with ROI drawn a few voxels away from the endocortical perimeter. Cortical ROIs in the femur midshaft were automatically selected using the periosteal perimeter as the outer boundary, whereas cortical ROIs for the distal femur were contoured manually to exclude trabecular bone. Segmentation was performed using a global threshold of 90–255. Architectural parameters were calculated using CTAn.

### Bone histomorphometry and serum markers of bone remodeling activity.

Tibias were decalcified, embedded in paraffin, longitudinally sectioned to prepare frontal sections (5 μm thickness), and stained with H&E for visualization of bone marrow adiposity, as described previously (56). Images were captured using an Olympus IX70 microscope with a camera (Qicam) or a Zeiss Axioscan 7 slide scanner. Bone marrow adipocyte area fraction (BMAd.Ar/M.Ar, %) and adipocyte density (N.BMAd/Ma.Ar, no./mm^2^), were quantified (Bioquant Osteo) as described previously (56). Femurs were dehydrated in a graded ethanol series, embedded in methylmethacrylate, and sectioned using an Isomet low-speed diamond saw. Periosteal (Ps) and endocortical (Ec) mineralizing surfaces (MS/BS, %), mineral apposition rates (MAR; microns/day), and bone formation rates (BFR/BS, microns/day) were calculated as described previously (56, 57). Trabecular bone analyses were not performed in the femur due to the scarcity of trabeculae remaining in aged female mice.

Bone remodeling activity was quantified in the serum via measurement of P1np (bone formation; Imnmunodiagnostic Systems, AC-33F1) and Tracp5B (bone resorption; Immunodiagnostic Systems, SB-TR103) following the manufacturer’s protocols. Absorbance values for ELISA plates were measured with a microplate reader (BioTek Cytation 5) using Gen5 software. Serum data were normalized to calibrants provided.

### Primary BMSC culture.

Primary BMSCs were harvested from the femur as described previously (14, 56–58). Cells were seeded into 12-well plates (Corning, 0720082) at 4 million cells/well in growth medium consisting of minimum essential medium α (MEM-α; Gibco/Thermo Fisher Scientific, 12561-072) with 20% fetal bovine serum (FBS; Corning, 35-010-CV), 1% antibiotic-antimycotic (Gibco, 15240–062), and 1% nonessential amino acids (Gibco, 11140-050). Cultures were fixed and stained with 0.5% crystal violet solution (Thermo Fisher Scientific, C581-25) on days 7 and 14 of culture to assess colony formation and cell density, where crystal violet–stained area and total well area were quantified (Bioquant Osteo). Additional cultures were subjected to osteogenic induction (to obtain primary BMSC-OBs) for which cells were seeded in 12-well plates at 4 million cells/well in growth medium supplemented with ascorbic acid (50 μg/mL; Sigma-Aldrich, A4544) and β-glycerophosphate (10 mM; Sigma-Aldrich, G9422). Culture medium was changed every 3–4 days. To assess mineralized matrix formation, BMSC-OB cultures were maintained for 21 days, fixed with 10% neutral-buffered formalin, and stained with alizarin red S (Sigma-Aldrich, A5533). Stained area normalized to total well area was quantified (Bioquant Osteo). Gene expression was assessed in BMSC-OB cultures on day 21. Cells were lysed in TRIzol (Thermo Fisher Scientific, 15-596-018). Total RNA was isolated, reverse transcribed as described previously (56), and cDNA was subjected to real-time PCR amplification (37.5 ng cDNA per 15 μL reaction volume reaction, run in triplicate) using a Bio-Rad CFX Connect system and SYBR green (Thermo Fisher Scientific, A25780). Gene expression levels were quantified using the comparative threshold cycle (2^−ΔΔCt^) method. Glyceraldehyde-3-phosphate dehydrogenase (Gapdh) was used as the internal control (housekeeping gene) for normalization. Primer sequences are listed in Supplemental Table 6.

### Bulk RNA-seq.

Following primary BMSC isolation, cortical bone shafts were digested with collagenase and frozen in liquid nitrogen. One TA per mouse (*n* = 4/group) and cortical bone shafts (*n* = 4/group) were subjected to bulk RNA-seq. For cortical bone shafts, RNA was isolated with TRIzol as described previously (56). Frozen muscle samples were sent to a commercial vendor (Medgenome) and total RNA was extracted using the Maxwell RSC simplyRNA Tissue kit (Promega, AS1340), following the manufacturer’s protocol. Samples were homogenized in the provided lysis buffer using a bead mill homogenizer and loaded onto the Maxwell RSC cartridge along with the necessary reagents for automated RNA extraction. Resulting RNA was quantified using a Qubit Flex Fluorometer (Invitrogen, Q33327) and assessed for integrity using an Agilent TapeStation 4200. Libraries were generated using the Stranded mRNA Prep kit (Illumina, 20040532), following the manufacturer’s protocol with 300 ng RNA input. The protocol was initiated by the selective purification of polyadenylated RNA molecules from total RNA using oligo-dT magnetic beads. Captured mRNA was fragmented using divalent cations under elevated temperature. First strand cDNA synthesis was performed using random hexamer primers and reverse transcriptase, with actinomycin D included to improve strand specificity. The RNA template was removed, and second strand cDNA synthesis was carried out using DNA polymerase I and RNase H. Strand specificity was maintained by incorporating dUTP in place of dTTP during second strand synthesis, enabling subsequent degradation of the second strand during post-PCR processing. The 3′ ends of the cDNA fragments were adenylated to prevent self-ligation, and adapters were ligated to prepare the cDNA for hybridization onto a flow cell. The adapter-ligated cDNA was enriched through PCR amplification and purified. Final library quality and quantity were assessed using a Qubit Flex Fluorometer (Invitrogen, Q33327) for concentration measurements and an Agilent TapeStation 4200 for size distribution analysis. Libraries were sequenced on an Illumina NovaSeq 6000 platform for 300 cycles according to manufacturer’s instructions. For cortical bone, 16 paired-end raw sequence reads files (in fastq format) from RNA samples were transferred to the Partek Flow server via SFTP yielding 8 RNA samples. Analyses were performed using the Partek Flow RNA-seq tool (https://www.illumina.com/products/by-type/informatics-products/partek-flow.html). The total reads were between 25 million and 28 million reads per sample, with an average QA/QC score of at least 35.10 per sample prealignment. After prealignment QA/QC check and base trimming, the sequencing reads were aligned to the mm39 genome using the STAR aligner. The alignment rate ranged from 59.90% to 90.72% per sample with postalignment QA/QC score of at least 35.42. For skeletal muscle, 16 paired-end raw sequence reads files (in fastq format) from RNA samples were transferred to the Partek Flow server via SFTP resulting in 8 RNA samples. The analysis was performed using the Partek Flow RNA-Seq tool. The total reads were between 25 million and 28 million reads per sample, with an average QA/QC score of at least 35.28 per sample before alignment. After prealignment QA/QC check and base trimming the sequencing reads were aligned to the mm39 genome using the STAR aligner. The alignment rate ranged from 48.31% to 55.80% per sample with a postalignment QA/QC score of at least 35.67. For both tissue samples, the aligned reads were quantified to annotation model mm39-emsembl-release-104 using the Partek E/M algorithm, followed by data normalization (CPM, offset, and log_2_ transformation) to generate sequence counts of mouse genes for downstream differential expression analysis. For differential analysis of the RNA-seq data, normalized gene counts were analyzed using the LIMMA-Voom approach in Partek Flow. Volcano plots for each comparison were generated using Partek Flow with the top 50 protein-coding genes (with the largest absolute fold changes) shown in the plot. For GSEA, differential expression analysis was performed following differential expression analysis using DESeq2 (R Bioconductor package, https://bioconductor.org/packages/release/bioc/html/DESeq2.html), with GSEA performed using the R Bioconductor package clusterProfiler (v3.14.3) (https://bioconductor.org/packages/release/bioc/html/clusterProfiler.html).

### Proteomics.

Proteins were extracted from 1 quadriceps per mouse (*n* = 4/group) using RIPA buffer. After measuring total protein concentration, 50 μg total protein was aliquoted and precipitated by adding 8 volumes of cold acetone and 1 volume of 100% trichloroacetic acid (TCA). Precipitated proteins were washed with cold acetone and air dried before reconstitution into 40 μL of 8 M urea in 50 mM Tris-HCl (pH 8). Reduction and alkylation of the cysteine residues were then performed with 10 mM DTT and 55 mM iodoacetamide, respectively, followed by the addition of 360 μL of 50 mM ammonium bicarbonate buffer to reduce the urea concentration to below 1 M. Protein samples were digested by trypsin (Pierce) at a 1:20 ratio (w/w) and incubated at 37°C overnight. Digested protein samples were cleaned using a C-18 micro-spin plate (Harvard Apparatus) before liquid chromatography–mass spectrometry (LC-MS) analysis. The LC-MS analysis was performed using an Orbitrap Fusion tribrid mass spectrometer (Thermo Fisher Scientific) connected to an Ultimate 3000 nano-UPLC system (Thermo Fisher Scientific). Briefly, the peptide samples were trapped and washed on a Pepmap100 C18 trap (5 μm, 0.3 × 5 mm) at 20 μL/min using 2% acetonitrile in water (with 0.1% formic acid) for 10 minutes and then separated on a Pepman100 RSLC C18 column (2.0 μm, 75 μm × 150 mm) using a gradient of 2% to 40% acetonitrile with 0.1% formic acid over 120 minutes at a flow rate of 300 nL/min and a column temperature of 40°C. Eluted peptides were introduced into Orbitrap Fusion MS via a nano-electrospray ionization (nano-ESI) source with a temperature of 300°C and spray voltage of 2000 V. The peptides were analyzed by data-dependent acquisition (DDA) in positive mode using Orbitrap MS analyzer for precursor scan at 120,000 FWHM from 400 to 2000 *m*/*z* and ion-trap MS analyzer for MS/MS scans in top speed mode (3-second cycle time) with dynamic exclusion settings (repeat count 1 and exclusion duration 15 seconds). Higher-energy collisional dissociation (HCD) was used as a fragmentation method with a normalized collision energy of 32%. The raw MS and MS/MS spectra were processed using the Proteome Discoverer software by Thermo Fisher Scientific (v1.4) and searched against the Uniprot database using the SequestHT search algorithm (precursor ion mass tolerance: 10 ppm, product ion mass tolerance: 0.6 Da, static carbamidomethylation of +57.021 Da of cysteine and dynamic oxidation for methionine [+15.995 Da]). The Percolator PSM validator algorithm was used to validate the peptide spectrum matching and estimate the false discovery rate to be less than 1% (*q* value) (59). Proteins unable to be identified based on the database search results alone were grouped to satisfy the principles of parsimony. A protein report was generated containing the identities and number of PSM for each protein group, which were further utilized for spectral counting based semiquantitative analysis. Pathway analysis was performed with ToppGene (https://toppgene.cchmc.org/) to identify and prioritize neighboring genes of the seeds in protein-protein interaction network based on functional similarity.

### Statistics.

Experiments comparing only 2 groups (e.g., BAY vs. VEH treatment) were analyzed with Student’s 2-tailed *t* tests, whereas experiments comparing 3 or more groups were analyzed with 1-way ANOVA with Fisher’s least significant difference (LSD) post hoc analysis where appropriate. When experiments were conducted with both male and female animals (e.g., CH-223191 studies), groups were compared with 2-way ANOVA (e.g., factor 1 = treatment, AhR antagonist or vehicle; factor 2 = sex, male or female). All statistical analyses were completed with JMP Pro v.18 (JMP Statistical Discovery, LLC, SAS Institute); a *P* value of less than 0.05 was considered statistically significant and *P* values from 0.05 to 0.1 were noted as trends. Statistical analyses for the transcriptional activity data were performed on Renilla-corrected firefly luciferase values and analyses for qPCR data were performed on ΔΔCT values. Data from in vitro studies are shown as the mean ± SEM of all biological replicates, where each replicate sample is indicated by a separate data point. Data from in vivo studies are represented in box-and-whisker plots showing the median (line in box), quartiles (box bounds), and outlier fences for each dataset (whiskers), where outlier fences represent first quartile – 1.5 × (interquartile range) and third quartile + 1.5 × (interquartile range), and each data point shown represents 1 mouse.

### Study approval.

Research was conducted according to guidelines provided by the NIH *Guide for the Care and Use of Laboratory Animals* (National Academies Press, 2011) under protocols approved by the Augusta University Institutional Animal Care and Use Committee.

### Data availability.

The data that support the findings of this study are available in public repositories (RNA-seq: NCBI GEO GSE299537; MS proteomics data: ProteomeXchange Consortium/PRIDE partner repository PXD065529), in the Supporting Data Values file, or are available from the corresponding author upon request.

## Author contributions

KY was responsible for data curation, formal analysis, investigation, writing the original draft, and reviewing and editing subsequent drafts. SV contributed to data curation, formal analysis, investigation, and review and editing of the manuscript. DWA participated in formal analysis, investigation, and manuscript review and editing. HB was involved in investigation and manuscript review and editing. LR contributed to investigation, formal analysis, and manuscript review and editing. A Tuladhar conducted investigation and manuscript review and editing. CD was responsible for methodology, investigation, and manuscript review and editing. JCS carried out formal analysis, investigation, and manuscript review and editing. KD contributed to investigation and manuscript review and editing. RP participated in data curation, formal analysis, methodology, investigation, and manuscript review and editing. A Tripathi contributed to investigation and manuscript review and editing. MAC was responsible for methodology, funding acquisition, and manuscript review and editing. RZ was involved in data curation, investigation, and manuscript review and editing. MHJ participated in formal analysis and manuscript review and editing. JC was responsible for data curation, formal analysis, investigation, writing the original draft, and manuscript review and editing. WBB contributed to investigation, methodology, and manuscript review and editing. CMI was responsible for funding acquisition, methodology, and manuscript review and editing. WDH contributed to funding acquisition, methodology, and manuscript review and editing. MWH was involved in conceptualization, formal analysis, funding acquisition, investigation, methodology, project administration, resources, supervision, and manuscript review and editing. SF contributed to conceptualization, formal analysis, funding acquisition, investigation, methodology, project administration, resources, supervision, and manuscript review and editing. MEML was responsible for conceptualization, data curation, formal analysis, funding acquisition, investigation, methodology, project administration, resources, supervision, validation, visualization, writing the original draft, and manuscript review and editing.

## Supplementary Material

Supplemental data

Supporting data values

## Version 1 07/15/2025

In-Press Preview

## Version 2 08/22/2025

Electronic publication
