# Supplementary material for: Inhibition of AhR improves cortical bone and skeletal muscle function via preservation of neuromuscular junctions
Source: JCI Insight. 2025 Jul 15;10(16):e192047. doi: 10.1172/jci.insight.192047 (PMC12406733; doi:10.1172/jci.insight.192047)
Supplement: Supplemental data [file jciinsight-10-192047-s157.pdf]

Supplemental Methods

BAY2416964 treatment of young male and female C57BL/6 mice

BAY2416964 was obtained from a commercial supplier (Targetmol #T10270). Male and female C57BL/6J mice (strain #00664, 16 weeks old; n=12/sex) were obtained from the Jackson Laboratory and allowed one week to acclimate to the facility. Mice were randomly allocated to receive treatment with vehicle (PBS+10%DMSO+45% PEG400; n=6/sex) or BAY2416964 (30 mg/kg body mass dosage; n=6/sex) via intraperitoneal injection 5 days per week for 4 weeks. Muscle endurance was assessed at baseline (prior to the onset of treatment) and in the 4th week of treatment via hang-time testing, and muscle grip strength was assessed in the 4th week of treatment (Bioseb BIO-GS3) immediately prior to sacrifice.

AhR target gene expression in old and young mice

Male C57BL/6J mice at 4- and 22-months of age were obtained from the National Institute on Aging. Tibialis anterior muscles were collected at sacrificed and total RNA was extracted with TRIzol. Expression levels of the AhR target gene Cyp1a1 were quantified using the comparative threshold cycle ($2-\Delta\Delta Ct$) method as previously described (Bensreti et al, Bone. 2023 Aug:173:116811. doi: 10.1016/j.bone.2023.116811). Primer sequences for Cyp1a1 were as follows: Cyp1a1 F: 5'-ACTCTTCCCTGGATGCCTTC-3', Cyp1a1 R: 5'-TGTGGCCCTTCTCAAATGTCC-3'.

Supplementary Figures and Tables

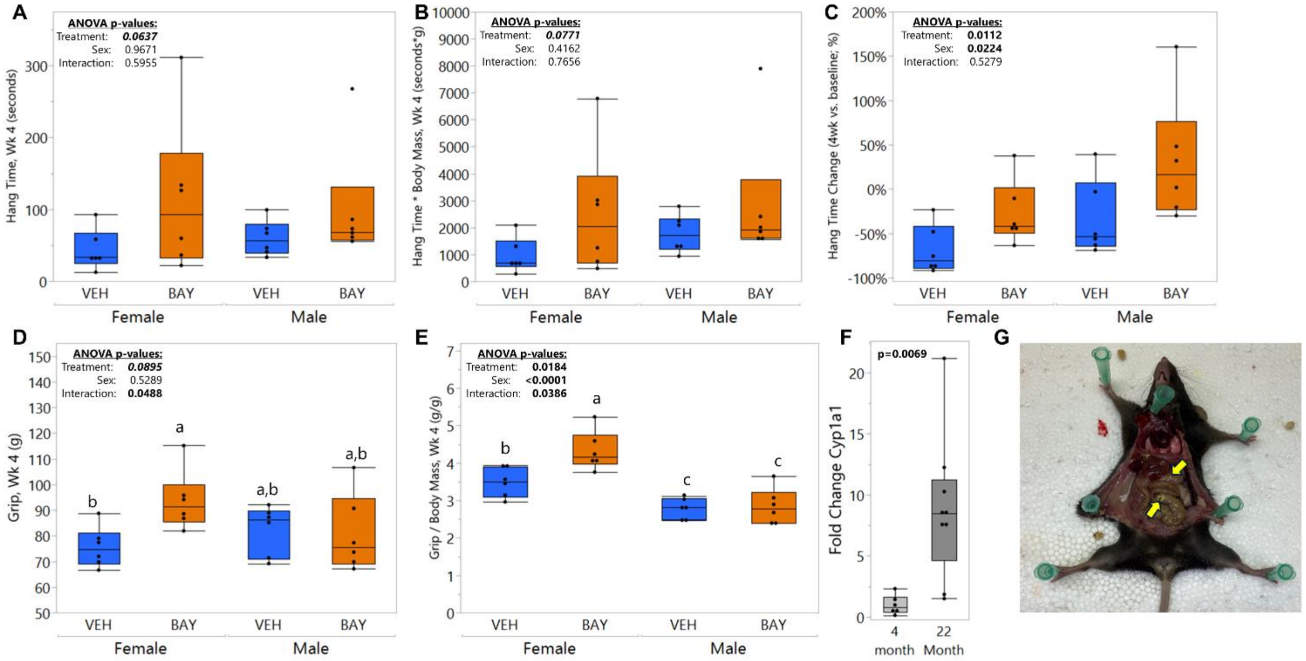

Supplemental Figure S1: BAY2416964 enhanced grip strength of young female but not male C57BL/6 mice. Hang time (**A**) and hang time normalized to body mass (**B**) tended to be higher in BAY- as compared to VEH-treated mice, and longitudinal analyses demonstrated a significant improvement in BAY-treated mice over the course of the study (**C**). Grip strength (**D**) and grip strength normalized to body mass (**E**) were enhanced by BAY treatment in female but not male mice. **F**) Gene expression levels of Cyp1a1 are significantly higher in tibialis anterior muscles from aged (22 month old) as compared to young (4 month old) mice, suggesting that the effect of AhR inhibition may be more pronounced in an aged model. **G**) At necropsy, evidence of BAY precipitation was noted in the peritoneal cavity (yellow arrows), reflecting the insoluble nature of this compound and suggesting that injection-based delivery modes requiring solubilization are not an optimal mode of delivery. Box plots show median, quartiles, and outlier fences for each group, where outlier fences represent first quartile −1.5* (interquartile range) and third quartile +1.5* (interquartile range), and each black circle represents one mouse. Two-way ANOVA p-values are shown on each graph, and groups with different superscript letters are significantly ($p<0.05$) different from one another as shown by post-hoc testing.

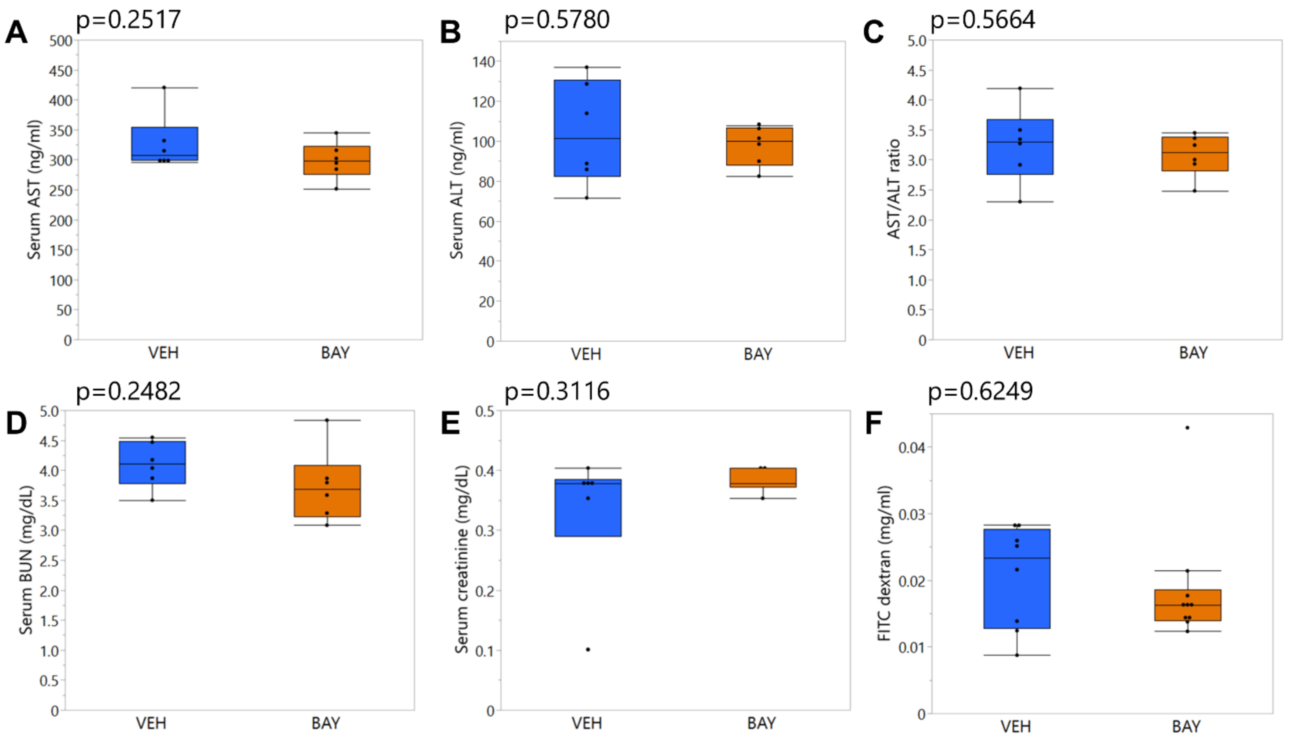

Supplemental Figure S2: BAY treatment did not impact liver, kidney, or gut function. Levels of **A**) aspartate aminotransferase (AST), **B**) alanine aminotransferase (ALT), C) AST/ALT ratio, **D**) blood urea nitrogen (BUN) and **E**) creatinine in the serum were unaffected by BAY treatment. Gut permeability, as shown by leakage of FITC-dextran into the circulation, was also not affected by BAY treatment (**F**). Box plots show median, quartiles, and outlier fences for each group, where outlier fences represent first quartile −1.5* (interquartile range) and third quartile +1.5* (interquartile range), and each black circle represents one mouse. P-values from t-tests comparing groups are shown above each graph.

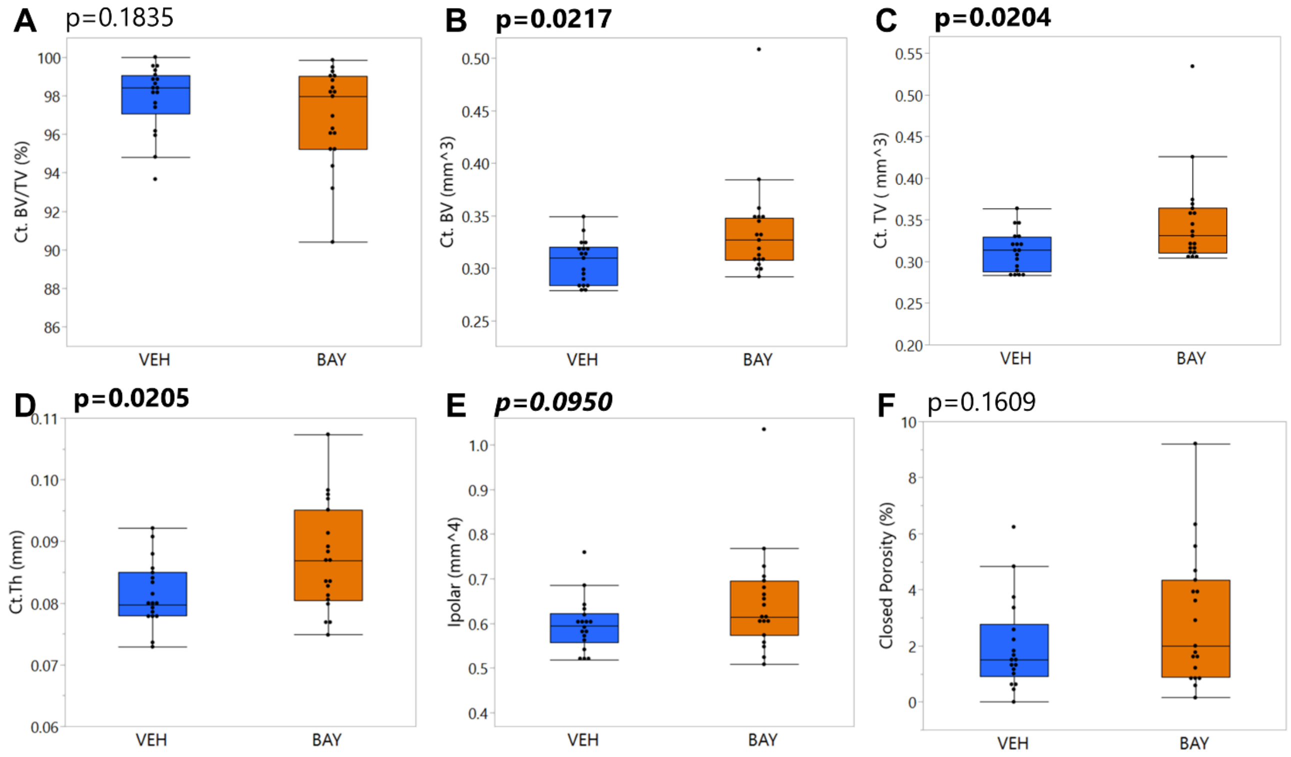

Supplemental Figure S3: Cortical bone in the distal femoral metaphysis was mildly improved by BAY treatment. MicroCT analyses of the distal femoral metaphysis cortex showed selective improvement in metrics of cortical bone mass in BAY-treated mice. While **A)** cortical bone volume fraction was not affected by treatment, **B)** cortical bone volume, **C)** cortical bone tissue volume, and **D)** cortical thickness were larger in BAY- as compared to VEH-treated mice. **E)** Polar moment of inertia showed a mild trend to be larger in BAY- as compared to VEH-treated mice, whereas **F) c**ortical bone porosity was not different between groups. Box plots show median, quartiles, and outlier fences for each group, where outlier fences represent first quartile −1.5* (interquartile range) and third quartile +1.5* (interquartile range), and each black circle represents one mouse. P-values from t-tests comparing groups are shown above each graph.

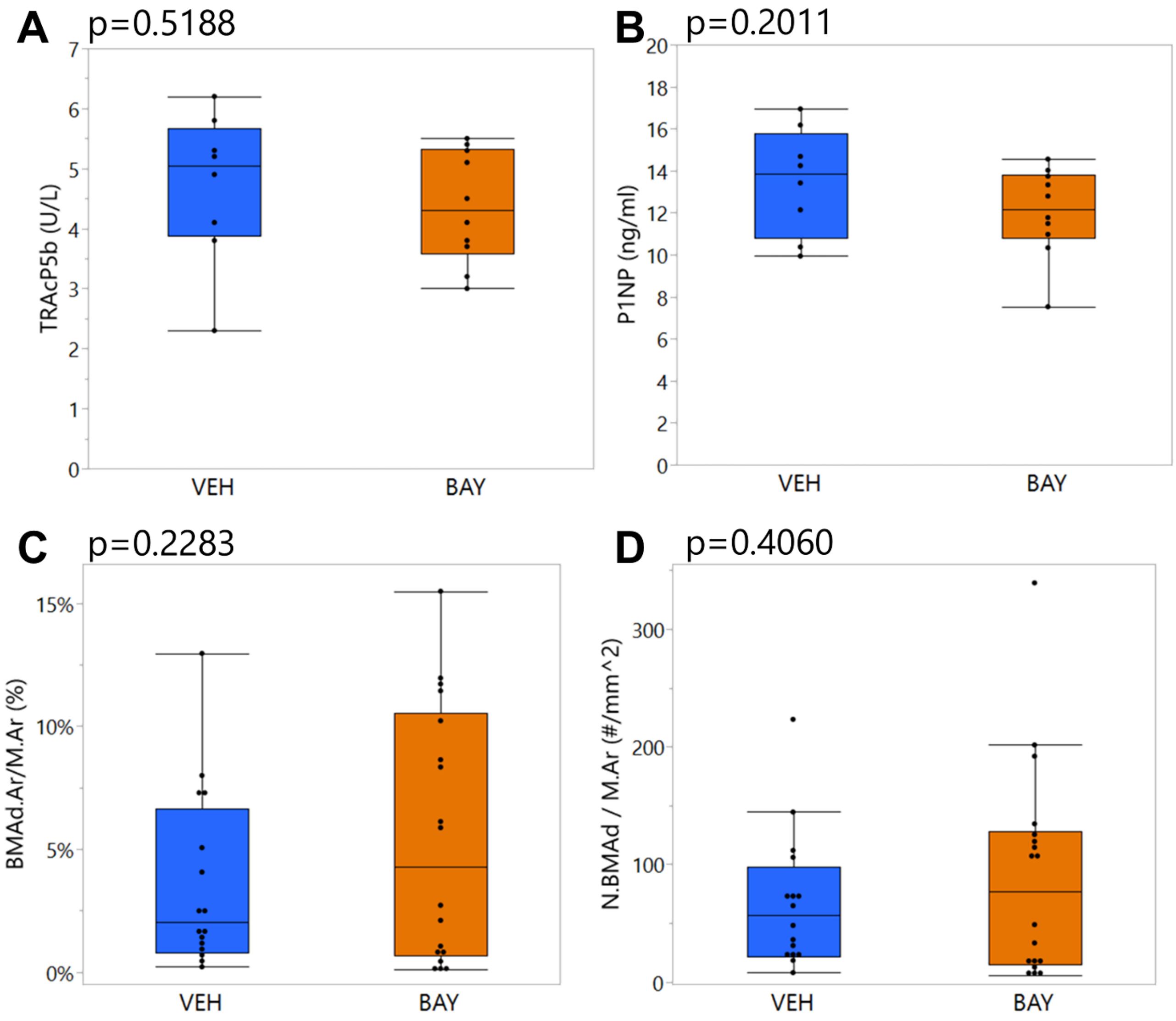

Supplemental Figure S4: Serum markers of bone remodeling and bone marrow adiposity were unaffected by BAY treatment. Box plots show median, quartiles, and outlier fences for each group, where outlier fences represent first quartile −1.5* (interquartile range) and third quartile +1.5* (interquartile range), and each black circle represents one mouse. P-values from t-tests comparing groups are shown above each graph.

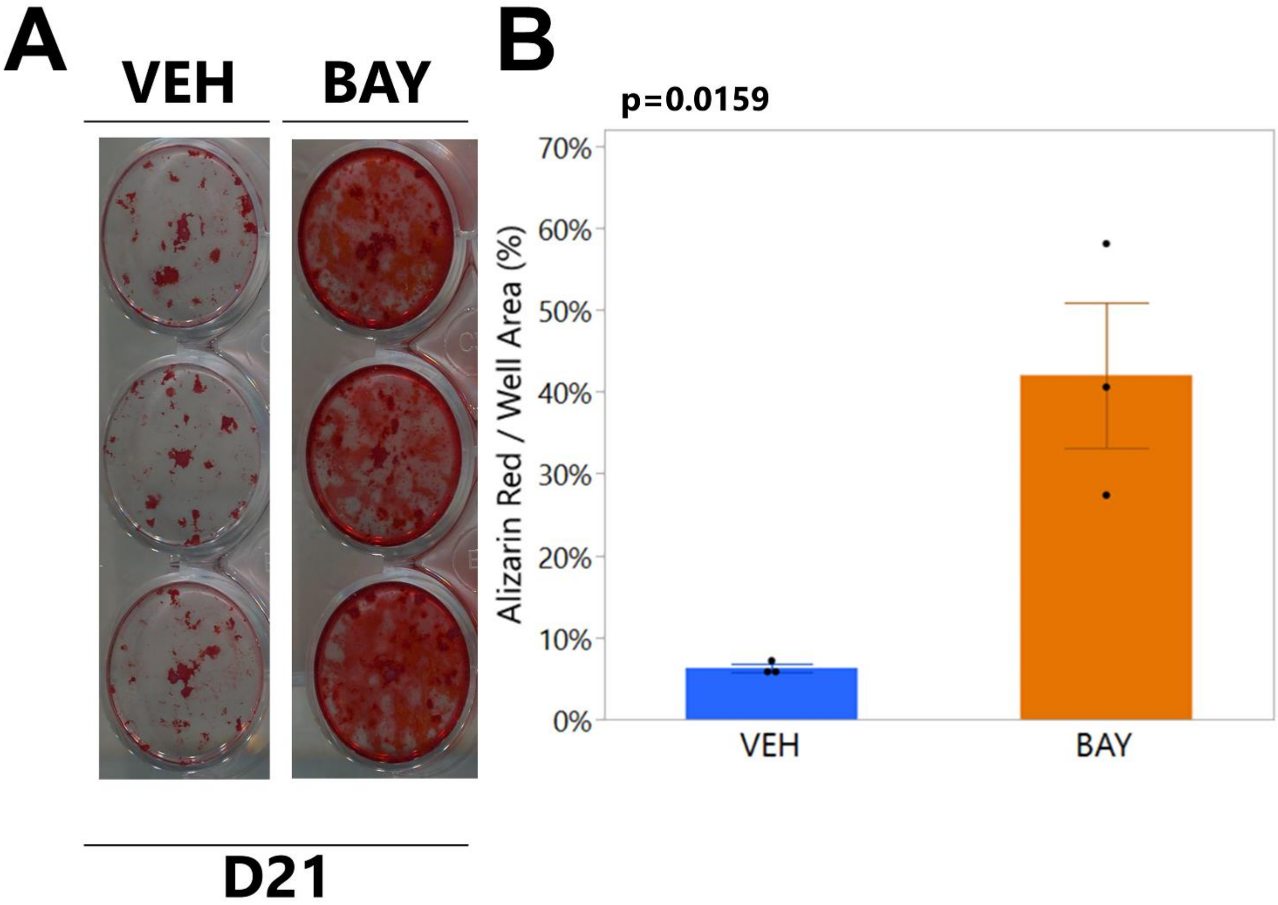

Supplemental Figure S5: **BMSC-derived osteoblasts from BAY-treated mice produced more matrix in vitro by day 21 (D21) in culture. A)** Alizarin red staining; each well represents one technical replicate culture. B) Quantification of alizarin red staining as alizarin red area normalized to total well area. Box plots show median, quartiles, and outlier fences for each group, where outlier fences represent first quartile −1.5* (interquartile range) and third quartile +1.5* (interquartile range), and each black circle represents one replicate culture. P-value from t-tests comparing groups is shown above the graph.

Supplemental Table 1: Cortical bone dynamic histomorphometry from the femur mid-diaphysis

Site	Property	VEH n=17			BAY n=18			P-value
Femur mid-diaphysis	Ps.MS/BS (%)	9.376	±	2.005	8.000	±	1.947	0.6247
	Ps.MAR (µm/day)	*unable to quantify*			*unable to quantify*			
	Ps.BFR/BS (µm/day)	*unable to quantify*			*unable to quantify*			
	Ec.MS/BS (%)	47.469	±	2.178	46.967	±	2.373	0.8876
	Ec.MAR (µm/day)	1.145	±	0.047	1.119	±	0.079	0.7791
	Ec.BFR/BS (µm/day)	0.543	±	0.032	0.529	±	0.049	0.8232

Means ± standard error are presented. VEH: vehicle, BAY: BAY2416964. One VEH-treated sample and one BAY-treated sample were lost during processing. Ps: Periosteal, Ec: Endocortical, MS/BS: mineralizing surface, MAR: mineral apposition rate, BFR/BS: bone formation rate (surface referent).

Supplemental Table 2: Inflammatory cytokines in serum and bone marrow supernatant fluid

Site	Property	VEH n=8			BAY n=10			P-value
Serum	IL-12 (pg/ml)	1.78	±	0.14	2.98	±	0.68	0.1390
	IL-1α (pg/ml)	6.53	±	0.87	6.02	±	0.76	0.6670
	IFN-γ (pg/ml)	7.51	±	2.78	9.21	±	2.02	0.6168
	TNF-α (pg/ml)	19.45	±	3.52	21.96	±	2.27	0.5422
	MCP1 (pg/ml)	24.67	±	2.56	21.12	±	1.88	0.2691
	IL-1β (pg/ml)	18.73	±	8.66	9.63	±	1.95	0.2713
	IL-10 (pg/ml)	55.37	±	8.87	44.71	±	6.20	0.3276
	IL-6 (pg/ml)	6.89	±	1.43	10.32	±	1.76	0.1635
	IL-27 (pg/ml)	166.38	±	29.56	234.27	±	62.71	0.3626
	IL-17α (pg/ml)	8.75	±	3.57	11.32	±	2.28	0.5368
	IFN-β (pg/ml)	148.96	±	63.02	170.20	±	45.64	0.7853
	GM-CSF (pg/ml)	2.68	±	0.77	3.62	±	1.25	0.5568
**Site**	**Property**	**VEH n=8**			**BAY n=9**			**P-value**
Bone marrow fluid	IL-12 (pg/ml)	0.95	±	0.22	0.80	±	0.11	0.5322
	IL-1α (pg/ml)	271.45	±	24.29	272.73	±	19.94	0.9679
	IFN-γ (pg/ml)	2.52	±	0.25	3.90	±	0.87	0.1711
	TNF-α (pg/ml)	7.44	±	0.87	6.46	±	0.52	0.3368
	IL-1β (pg/ml)	11.44	±	0.71	10.08	±	0.75	0.2109
	IL-10 (pg/ml)	8.51	±	2.16	12.39	±	2.31	0.2429
	IL-6 (pg/ml)	7.74	±	0.99	7.78	±	0.78	0.9726
	IL-17α (pg/ml)	1.13	±	0.15	0.88	±	0.13	0.2396

Means ± standard error are presented. VEH: vehicle, BAY: BAY2416964. IL: interleukin. IFN-γ: interferon gamma, TNF-α: tumor necrosis factor alpha, MCP-1: Monocyte chemoattractant protein-1, IFN-β: interferon beta, GM-CSF: granulocyte macrophage colony stimulating factor.

Supplemental Table 3: Trabecular bone architecture in the distal femoral metaphysis and spine

Site	Property	VEH n=18			BAY n=19			P-value
Distal femur	Tb.BV/TV (%)	1.713	±	1.300	0.545	±	0.169	0.3660
	Tb.Th (mm)	0.045	±	0.005	0.046	±	0.005	0.8664
	Tb.Sp (mm)	0.441	±	0.020	0.456	±	0.003	0.4494
	Tb.N (1/mm)	0.369	±	0.300	0.089	±	0.025	0.3469
Lumbar vertebra	Tb.BV/TV (%)	9.222	±	1.194	9.287	±	0.587	0.9607
	Tb.Th (mm)	0.052	±	0.001	0.052	±	0.001	0.8013
	Tb.Sp (mm)	0.428	±	0.022	0.421	±	0.014	0.7882
	Tb.N (1/mm)	1.842	±	0.285	1.786	±	0.107	0.8505

Means ± standard error are presented. VEH: vehicle, BAY: BAY2416964. Tb BV/TV: trabecular bone volume fraction, Tb.Th: trabecular thickness, Tb.Sp: trabecular separation, Tb.N: trabecular number.

Supplemental Table 4: Cortical bone enriched pathways identified from RNAseq analyses

**Description**	**Enrichment score**	**P-value**
Olfactory transduction	58.25	5.02E-26
Neuroactive ligand-receptor interaction	25.65	7.22E-12
Phototransduction	7.24	7.20E-04
Nicotine addiction	6.43	1.61E-03
Cholinergic synapse	3.99	1.86E-02
Glycosphingolipid biosynthesis - globo and isoglobo series	3.61	2.71E-02
Cell adhesion molecules	3.41	3.30E-02
Retinol metabolism	3.32	3.62E-02
Ascorbate and aldarate metabolism	3.25	3.89E-02
Metabolism of xenobiotics by cytochrome P450	3.05	4.72E-02
ABC transporters	*2.73*	*6.51E-02*
Pentose and glucuronate interconversions	*2.70*	*6.73E-02*
Tryptophan metabolism	*2.67*	*6.96E-02*
Steroid hormone biosynthesis	*2.59*	*7.48E-02*
Inflammatory mediator regulation of TRP channels	*2.55*	*7.82E-02*
Vitamin digestion and absorption	*2.38*	*9.29E-02*
GABAergic synapse	*2.36*	*9.40E-02*

Supplemental Table 5: Skeletal muscle enriched pathways identified from RNAseq analyses

**Description**	**Enrichment score**	**P-value**
Circadian entrainment	8.13	2.95E-04
Morphine addiction	7.36	6.35E-04
Human immunodeficiency virus 1 infection	6.79	1.13E-03
Graft-versus-host disease	6.53	1.46E-03
Allograft rejection	6.10	2.25E-03
Autoimmune thyroid disease	6.10	2.25E-03
GABAergic synapse	5.74	3.21E-03
Type I diabetes mellitus	5.71	3.30E-03
Olfactory transduction	5.67	3.44E-03
Viral myocarditis	5.25	5.26E-03
Amphetamine addiction	5.10	6.11E-03
Cell adhesion molecules	4.63	9.72E-03
Cholinergic synapse	4.55	1.06E-02
Human cytomegalovirus infection	4.07	1.70E-02
Ras signaling pathway	3.90	2.03E-02
Chemical carcinogenesis - DNA adducts	3.81	2.21E-02
Serotonergic synapse	3.74	2.37E-02
Drug metabolism - other enzymes	3.70	2.47E-02
Dopaminergic synapse	3.59	2.77E-02
Rheumatoid arthritis	3.58	2.78E-02
Systemic lupus erythematosus	3.47	3.11E-02
Metabolism of xenobiotics by cytochrome P450	3.41	3.32E-02
Retrograde endocannabinoid signaling	3.34	3.56E-02
PI3K-Akt signaling pathway	3.30	3.68E-02
Chemokine signaling pathway	3.20	4.07E-02
MAPK signaling pathway	3.18	4.17E-02
Pathways in cancer	3.11	4.48E-02
Apelin signaling pathway	3.10	4.49E-02
Osteoclast differentiation	3.00	4.98E-02
Glutamatergic synapse	3.00	4.98E-02
Kaposi sarcoma-associated herpesvirus infection	*2.98*	*5.08E-02*
Steroid hormone biosynthesis	*2.71*	*6.65E-02*
Complement and coagulation cascades	*2.67*	*6.91E-02*
Epstein-Barr virus infection	*2.65*	*7.04E-02*
Glycosphingolipid biosynthesis - lacto and neolacto series	*2.63*	*7.23E-02*
PD-L1 expression and PD-1 checkpoint pathway in cancer	*2.55*	*7.84E-02*
Alcoholism	*2.51*	*8.12E-02*
Protein digestion and absorption	*2.41*	*9.01E-02*
Viral protein interaction with cytokine and cytokine receptor	*2.39*	*9.16E-02*
Basal cell carcinoma	*2.31*	*9.97E-02*
VEGF signaling pathway	*2.31*	*9.97E-02*

Supplemental Table 6. Primer sequences for PCR reactions

Primer	Direction	Sequence
Gapdh	Forward	5’-GGGAAGCCCATCACCATC-3’
Gapdh	Reverse	5’-GCCTCACCCCATTTGATGTT-3’
Ccnd1	Forward	5’- AGAAGGAGATTGTGCCATCCA-3’
Ccnd1	Reverse	5’- CTCACAGACCTCCAGCATCCA-3’
Runx2	Forward	5’-GGCACAGACAGAAGCTTGATGA-3’
Runx2	Reverse	5’-GAATGCGCCCTAAATCACTGA-3’
Col1a1	Forward	5’-GCTTCACCTACAGCACCCTTGT-3’
Col1a1	Reverse	5’-TGACTGTCTTGCCCCAAGTTC-3’
Alpl	Forward	5’- CACAGATTCCCAAAGCACCT-3’
Alpl	Reverse	5’- GGGATGGAGGAGAGAAGGTC-3’
